# Give or Take: Effects of Electron-Accepting/-Withdrawing Groups in Red-Fluorescent BODIPY Molecular Rotors

**DOI:** 10.3390/molecules27010023

**Published:** 2021-12-21

**Authors:** Karolina Maleckaitė, Domantas Narkevičius, Rugilė Žilėnaitė, Jelena Dodonova-Vaitkūnienė, Stepas Toliautas, Sigitas Tumkevičius, Aurimas Vyšniauskas

**Affiliations:** 1 Center of Physical Sciences and Technology, Saulėtekio av. 3, LT-10257 Vilnius, Lithuania; karolina.maleckaite@ftmc.lt; 2 Institute of Chemistry, Faculty of Chemistry and Geosciences, Vilnius University, Naugarduko str. 24, LT-03225 Vilnius, Lithuania; domantas.narkevicius@chgf.stud.vu.lt (D.N.); rzilenaitei@gmail.com (R.Ž.); jelena.dodonova@gmail.com (J.D.-V.); sigitas.tumkevicius@chf.vu.lt (S.T.); 3 Institute of Chemical Physics, Faculty of Physics, Vilnius University, Saulėtekio av. 9-III, LT-10222 Vilnius, Lithuania; stepas.toliautas@ff.vu.lt

**Keywords:** fluorescent probes, photophysics, molecular rotors, microviscosity, polarity sensing

## Abstract

Mapping microviscosity, temperature, and polarity in biosystems is an important capability that can aid in disease detection. This can be achieved using fluorescent sensors based on a green-emitting BODIPY group. However, red fluorescent sensors are desired for convenient imaging of biological samples. It is known that phenyl substituents in the β position of the BODIPY core can shift the fluorescence spectra to longer wavelengths. In this research, we report how electron-withdrawing (EWG) and -donating (EDG) groups can change the spectral and sensory properties of β-phenyl-substituted BODIPYs. We present a trifluoromethyl-substituted (EWG) conjugate with moderate temperature sensing properties and a methoxy-substituted (EDG) molecule that could be used as a lifetime-based polarity probe. In this study, we utilise experimental results of steady-state and time-resolved fluorescence, as well as quantum chemical calculations using density functional theory (DFT). We also explain how the energy barrier height ($E_{a}$) for non-radiative relaxation affects the probe’s sensitivity to temperature and viscosity and provide appropriate $E_{a}$ ranges for the best possible sensitivity to viscosity and temperature.

## 1. Introduction

Fluorophores that are sensitive to environmental properties are very useful in biological studies and help to understand changes in the intracellular environment. Fluorescent molecular probes are widely used for imaging polarity [1,2], temperature [3,4], and microviscosity [5,6,7]. Fluorescent sensors have already been utilised in experimental objects, such as live cells [8,9,10], various organelles [3,11,12], polymers [13,14], aerosols [15,16], and lipid membranes [17,18,19]. The working principle of the majority of such sensors is based on the competition between fluorescence and non-radiative relaxation, where the rate of the latter is heavily affected by either viscosity, temperature, or polarity [20]. Together with fluorescence microscopy or fluorescence lifetime imaging microscopy (FLIM), fluorescent probes provide a non-invasive method for imaging changes in the medium [11,21,22].

**Figure 1:**
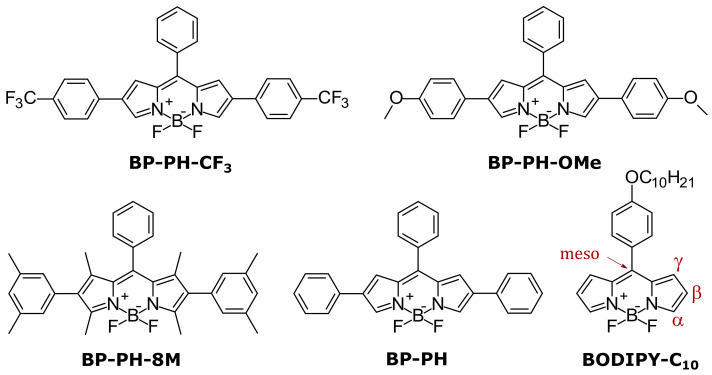
Molecular structures of BODIPYs investigated in this research (BP-PH-CF_3_, BP-PH-OMe, BP-PH-8M) together with previously reported BP-PH [33] and a well-known viscosity probe BODIPY-C_10_.

Some of the most popular microviscosity probes are based on the BODIPY group [8,23,24], such as BODIPY-C_10_ (Figure 1). They are generally used as fluorescence lifetime probes rather than simple fluorescence intensity probes due to the independence of lifetime on local concentration of fluorophores and the conditions of excitation and detection [25]. These probes stand out for having a monoexponential fluorescence decay, which simplifies data analysis [26]. Studies about polarity- and temperature-sensitive BODIPY-based fluorophores have also been published [9,27,28]. Furthermore, it has already been shown that a single BODIPY fluorophore can be partially sensitive to all three parameters: polarity, temperature, and microviscosity [29]. All this indicates that the BODIPY group is a perfect platform for the development of all mentioned types of environmental sensors.

However, since a bare BODIPY fluorophore absorbs and emits close to 500 nm [21], the majority of BODIPY-based sensors are not red absorbers or emitters. Creating a red-fluorescent sensor is an important task because such a sensor would prevent the light scattering in deep tissue imaging and avoid an overlap with autofluorescence [30,31]. Moreover, it would enable scientists to use a red BODIPY probe together with other fluorophores for various markings in live cells [32] that often tend to be green, such as green fluorescent protein (GFP). Recently, we published an article on a red fluorescent β-phenyl-substituted BODIPY microviscosity sensor [33]. We decided to explore this group of BODIPY derivatives with β-phenyls more thoroughly (Figure 1) and to continue aiming for better microviscosity, temperature, and polarity probes.

In this research, we present how the addition of an electron-withdrawing group (EWG) or an electron-donating group (EDG) to β-phenyls allows the tuning of the absorption and fluorescence spectra of the fluorophores. Furthermore, we investigate the sensitivity of the new probes to viscosity, temperature, and solvent polarity. The results show that attaching EDG to β-phenyls can suppress the temperature and viscosity sensitivity but enhance the polarity sensitivity, leading to a reliable red polarity probe. Finally, by performing theoretical calculations, we demonstrate that the extent of viscosity or temperature sensitivity is heavily determined by the energy barrier for non-radiative relaxation. We suggest that the acceptable barrier height for a viscosity sensor should not exceed 100 meV, whereas, for a temperature probe, it should fall within 100–200 meV.

## 2. Results and Discussion

### 2.1. Absorbance and Fluorescence Spectra

**Figure 2:**
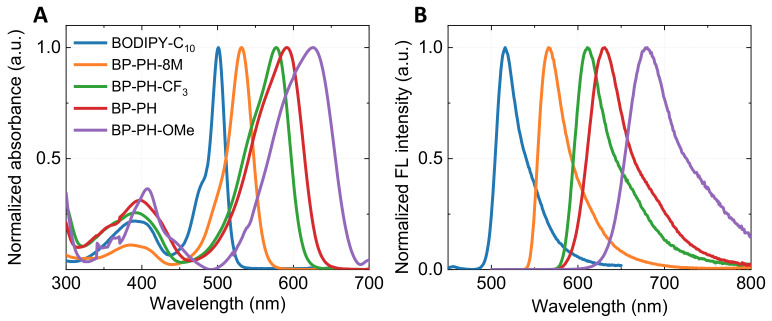
Absorbance (**A**) and fluorescence emission (**B**) spectra of BODIPY-C_10_ (blue), BP-PH-8M (orange), BP-PH-CF_3_ (green), BP-PH (red), and BP-PH-OMe (purple).

We begin by investigating basic spectroscopic properties of the new fluorophores and compare them to those of BP-PH and BODIPY-C_10_. The absorbance spectra (Figure 2A) of all dyes show a higher energy band at 300–450 nm and the main absorption band in the 500–630 nm region. BP-PH shows an increased absorption wavelength (Δλ = 90 nm) compared to the well-studied BODIPY-C_10_ due to the extension of conjugation. The addition of EWG in β-phenyls (BP-PH-CF_3_) results in a small blue-shift of the main absorption band compared to BP-PH (Δλ = 15 nm). An opposite effect is observed when the EDG is attached to β-phenyls (BP-PH-OMe), resulting in the highest absorption wavelength of 625 nm. The position of the main absorption band of the new derivative BP-PH-8M is blueshifted by 60 nm in contrast to previously reported BP-PH. The shift is caused by the addition of the methyl group in β-phenyls and the BODIPY core, which restricts the conjugation in BP-PH-8M.

Very similar blue- and red-shift tendencies are observed in the fluorescence spectra (Figure 2B). BP-PH-8M and BP-PH-CF_3_ correspond to the shorter wavelengths with the fluorescence maximum at 565 nm and 610 nm, respectively. In contrast, BP-PH-OMe shows the largest bathochromic shift, with the fluorescence peak at 680 nm due to an introduction of the EDG. A Stokes shift for unreported derivatives BP-PH-8M, BP-PH-CF_3_, and BP-PH-OMe was 1169 cm${}^{-1}$, 998 cm${}^{-1}$, and 1294 cm${}^{-1}$, respectively. The results also show that EWG at β-phenyls decreases the Stokes shift, while the opposite happens when EDG is introduced. As a result, by varying substituents on the β-phenyls, we were able to tune the emission wavelengths of new BODIPY fluorophores over a 500–700 nm range. In addition, quantum yield (QY) measurements in toluene demonstrated that attaching EWG to β-phenyls increases the QY value by 29% (BP-PH-CF_3_), while EDG reduces it by 16% (BP-PH-OMe), with respect to previously reported BP-PH without β-phenyl substitutes [33]. BP-PH-8M showed the highest QY of 87% due to restricted intramolecular rotation of β-phenyls. The absorption, fluorescence emission, Stokes shift, fluorescence lifetime, QY, and radiative and non-radiative relaxation values of the investigated conjugates are displayed in Table 1. Normalised absorbance and fluorescence spectra of BP-PH-8M and BP-PH-CF_3_ in solvents of various polarities are shown in Figure S1, ESI.

### 2.2. Theoretical Calculations

The DFT calculations correctly predict the trend of increasing absorption and fluorescence wavelengths from BODIPY-C_10_ to BP-PH-OMe (Table 1). The geometry of BP-PH-8M shows that the methyl groups on the BODIPY core force β-phenyls out of plane (Figure S2, ESI), leading to weaker conjugation and shorter absorption and fluorescence wavelengths. Furthermore, the DFT calculations reveal that increasing absorption and fluorescence wavelengths, going from BP-PH-CF_3_, to BP-PH, to BP-PH-OMe, are the result of a closer energy match between HOMO of BODIPY and β-phenyls (Figure S3, ESI). As a result, the HOMO of the resulting molecule is higher in energy, leading to a smaller HOMO-LUMO gap and higher absorption and fluorescence wavelengths. We note that theoretical wavelengths are shorter than experimental wavelengths by approximately 100 nm. This is a result of a well-known weakness of DFT, resulting in the overestimation of electronic transition energies in BODIPY fluorophores [34,35,36].

**Figure 3:**
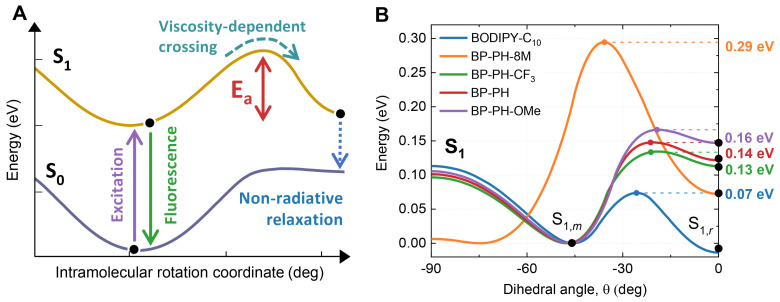
(**A**) Theoretical model of deexcitation pathways in BODIPY-based molecular rotors. (**B**) Potential energy surface curves of the first excited electronic state (S_1_) of BODIPY-C_10_ (blue), BP-PH-8M (orange), BP-PH-CF_3_ (green), BP-PH (red), and BP-PH-OMe (purple) calculated using DFT. θ is a dihedral angle between the BODIPY core and the *meso*-phenyl group. The S_1,m_ minima of curves were set to 0 for easier comparison.

*Meso*-phenyl BODIPYs are known for their viscosity and temperature sensitivity, which arises due to the competition between fluorescence and non-radiative relaxation [25]. The key factor affecting the rate of non-radiative relaxation is the height of energy barrier that the molecule needs to cross during the rotation of *meso*-phenyl in order to relax non-radiatively (Figure 3A) [37,38,39,40]. Therefore, we calculated the barriers for the new BODIPY compounds and contrasted them with the barriers for BODIPY-C_10_ and BP-PH (Figure 3B) [33]. BP-PH-8M has by far the highest barrier due to extra methyl groups that prevent the rotation of *meso*-phenyl. This explains why BP-PH-8M has the highest quantum yield of fluorescence and the slowest non-radiative decay rate (Table 1). The remaining molecules have smaller barriers, resulting in a faster non-radiative relaxation.

### 2.3. Time-Resolved Fluorescence and Its Sensitivity to Viscosity, Temperature and Polarity

**Figure 4:**
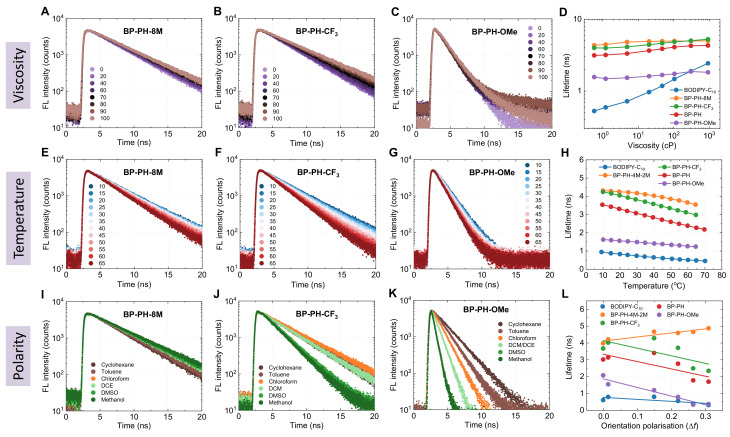
Time-resolved fluorescence decays of BP-PH-8M, BP-PH-CF_3_, BP-PH-OMe, and their sensitivity to viscosity, temperature, and polarity. (**A**–**D**) Viscosity dependence in toluene-castor oil mixtures. (**E**–**H**) Temperature dependence obtained in toluene. (**I**–**L**) Polarity dependence in different polarity solvents. The polarity of solvents is quantified using their orientational polarizability. The data for BP-PH and BODIPY-C_10_ is provided for comparison in (**D**,**H**,**L**).

In order to test if the new molecules could be used as probes of their environment, we explored their viscosity, temperature, and polarity sensing capabilities. Viscosity sensitivity measurements were performed in non-polar toluene/castor oil mixtures, covering the viscosity range of 0.5–920 cP. The majority of the observed fluorescence decays were monoexponential. The average lifetimes of biexponential decays were calculated using Equation (7) (Materials and Methods section), owing to the small contribution from the self-fluorescent castor oil. The viscosity-dependent fluorescence decays showed a slight viscosity dependence for BP-PH-CF_3_ (Figure 4B) and almost no viscosity dependence for BP-PH-8M or BP-PH-OMe (Figure 4A,C). Overall, the new derivatives showed much lower viscosity sensitivity compared to the viscosity sensor BODIPY-C_10_ (Figure 4D). This is expected, as theoretically calculated energy barriers for viscosity-sensitive non-radiative relaxation are larger than those for BODIPY-C_10_. Thus, these results support the hypothesis that a large energy barrier predicted by the DFT calculations leads to little to no viscosity sensitivity [33,41]. Conjugates BP-PH-8M and BP-PH-CF_3_ showed very similar fluorescence lifetimes, in a 3–5 ns range, to the previously reported conjugate without additional moieties (BP-PH). Meanwhile, BP-PH-OMe showed much shorter lifetimes, around 1.5 ns.

Temperature-dependent fluorescence decays recorded in toluene reveal that all new fluorophores exhibited moderate temperature dependence (Figure 4E–G). The extent of temperature dependence was similar to BP-PH, although new conjugates BP-PH-8M and BP-PH-CF_3_ showed longer lifetimes. BP-PH-OMe showed the weakest temperature sensitivity; its small lifetime values were more comparable to the widely studied BODIPY-C_10_, which showed low lifetimes in the low viscosity solvent (toluene). Fits of Figure 4H are shown in Figure S4, ESI.

Lastly, the polarity dependence experiments were performed (Figure 4I–L). BP-PH-8M stood out among all the fluorophores; its sensitivity to solvent polarity was minimal (Figure 4I). The key structural difference of BP-PH-8M compared to other fluorophores was the existence of the methyl groups that prevented the rotation of the *meso*-phenyl group (Figure 1). Therefore, it is likely that the non-radiative relaxation pathway responsible for the polarity sensitivity involves the rotation of the *meso*-phenyl substituent. This particular intramolecular rotation is known to result in non-radiative relaxation of *meso*-phenyl-BODIPYs [42]. However, it also causes viscosity-sensitivity [25], which BP-PH-OMe does not possess. Therefore, another non-radiative relaxation pathway is likely to also involve the rotation of the *meso*-phenyl group.

**Figure 5:**
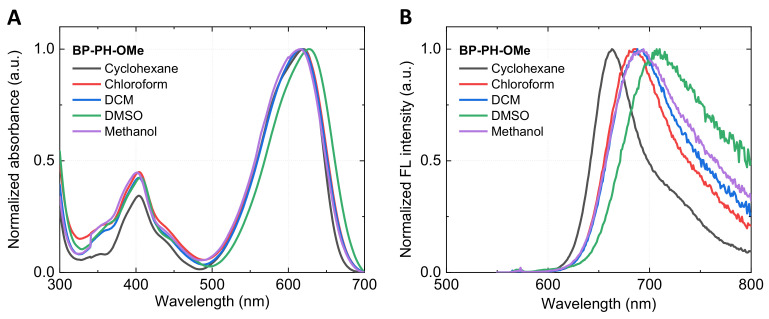
Absorbance (**A**) and fluorescence emission (**B**) spectra of BP-PH-OMe in various polarity solvents. From non-polar to very polar: cyclohexane (dark grey), chloroform (red), dichloromethane (DCM; blue), dimethyl sulfoxide (DMSO; green), and methanol (purple).

BP-PH-CF_3_ with the EWG substitute showed moderate polarity-sensitive properties. The kinetics of trifluoromethyl-substituted BODIPY (Figure 4J) was split into two groups: one consisted of non-polar and medium-polar solvents (cyclohexane, toluene, chloroform, and DCM) and the other had very polar solvents (DMSO and methanol). Meanwhile, EDG-substituted BP-PH-OMe showed strong polarity dependence and gradually decreasing lifetimes with increasing solvent polarity (Figure 4K). Thanks to this polarity dependence, the methoxy-substituted conjugate could be used as a red-emitting lifetime-based polarity sensor. Compared to the absolute majority of other known fluorescent polarity sensors, such as the Reichardt’s dye [43], BP-PH-OMe showed a far smaller solvatochromic shift (Figure 5). However, the constant spectral position of BP-PH-OMe (Figure 5) is an advantage if the FLIM technique is used. The spectral detection window can be correctly chosen beforehand without the need to guess, while the polarity can be determined from the fluorescence lifetime of BP-PH-OMe.

The visually observed trends (Figure 4D,H,L) can be quantified using the relative sensitivity *S* [44]:

(1) $$S=-\frac{|\frac{\delta \tau }{\delta x}|}{\tau }\cdot 100\%\;,$$

where τ is a fluorescence lifetime, δ*x* is a change of the parameter (temperature—°C, polarity—Δ*f*). The *S* is expressed as a percentage change of lifetime per step change of the parameter. The calculated values of relative sensitivity to temperature and polarity are shown in Table 2. The higher the value, the stronger the sensitivity of the fluorophore to the particular environmental parameter. The sensitivity to viscosity is usually quantified using the *x* value from the Förster-Hoffmann equation [45]:

(2) $$\tau =C\eta^{x}\;,$$

where τ is fluorescence lifetime, η is viscosity, and *C*, *x* are constants. Unsurprisingly, a widely used viscosity sensor BODIPY-C_10_ showed the highest value of the viscosity sensitivity (*x* = 0.21, Figure S5, ESI). The remaining molecules displayed very minor viscosity sensitivity (≤0.05). All five conjugates showed small-to-moderate temperature sensitivity as a percentage change in lifetime per one degree Celsius, which was in the 0.3–1.0% range. However, a significant polarity sensitivity was observed for BP-PH-CF_3_, BP-PH, and BP-PH-OMe, the latter showing the strongest sensitivity to polarity (265%). Therefore, our results show that BODIPY compounds with β-phenyl substituents can be tuned for making new temperature and polarity sensors.

### 2.4. The Role of Energy Barrier for Determining Sensitivity to Viscosity and Temperature

In addition to the BODIPY fluorophores investigated in this work, there are a number of other known BODIPY probes that are sensitive to viscosity and temperature [9,10,21,28]. It is already known that the energy barrier for non-radiative relaxation is the key parameter affecting viscosity and temperature sensitivity [10,40,41]. Therefore, we set out to find optimal values that the energy barrier must have in order for the molecule to be the best sensor of viscosity or temperature. We started first with the viscosity probes.

**Figure 6:**
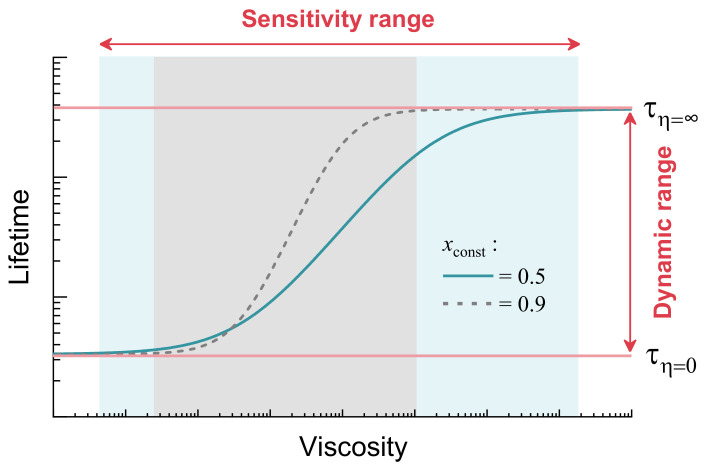
Fluorescence lifetime as a function of viscosity when the Förster–Hoffmann constant *x* = 0.5 (solid blue line) is at room temperature. If *x* is increased (*x* = 0.9, dashed grey line), the viscosity sensitivity becomes greater but the sensitivity range is decreased. Red lines indicate lifetime limits when the viscosity is 0 (bottom line, τ_η=0_) and infinity (top line, τ_η=*∞*_).

The fluorescence lifetime and intensity of viscosity-sensitive fluorophores typically show a sigmoidal dependence on viscosity on a double logarithmic plot, as shown in Figure 6, when the fluorophore is characterised over a sufficiently large viscosity range [29,41]. If the fluorophore is characterised only at intermediate viscosities, a linear viscosity-fluorescence lifetime (or intensity) dependence is observed on a logarithmic plot [24,46], which conforms to the Förster–Hoffmann equation (Equation (2)). Deviations from linearity occur due to the fact that a fluorophore cannot have a fluorescence lifetime equal to zero or infinity. The maximum possible fluorescence lifetime is set by a radiative decay constant, while the minimum possible lifetime is limited by the time required for the molecule to change its geometry and relax to ground state at zero viscosity and infinite temperature. The full sigmoidal viscosity-lifetime dependence is described by Equation (3), which was derived using the Förster–Hoffmann equation (Equation (2)) as a starting point [29]:

(3) $$\tau =\frac{1}{\frac{1}{C\eta^{x}+\frac{1}{k_{nr,max}}}\cdot e^{-\frac{E_{a}}{kT}}+k_{r}+k_{x}}\approx \frac{1}{\frac{1}{C\eta^{x}+\tau_{min}}\cdot e^{-\frac{E_{a}}{kT}}+\frac{1}{\tau_{max}}}\;,$$

where τ is a fluorescence lifetime, η is the dynamic viscosity, *C* and *x* are constants, *E_a_* is the activation energy for non-radiative relaxation, *k_nr,max_* is the non-radiative decay constant at zero viscosity and infinite temperature, *k* is the Boltzmann’s constant, *T* is the temperature in Kelvin, *k_r_* is the radiative decay constant, *k_x_* is the the sum of any other viscosity- and temperature-independent rate constants that lead to the population loss from the fluorescent state, and τ_min_ and τ_max_ are the minimum and maximum fluorescence lifetimes of the probe, respectively.

Usually, it is assumed that the parameter *x*, which comes from the Förster–Hoffman equation, is the most important parameter that shows sensitivity of the molecule to viscosity [25,26,29,46,47]. However, a high parameter *x* has disadvantages. For instance, increasing it from 0.5 to 0.9 (Figure 6) shortens the viscosity sensitivity range. This creates a viscosity probe that can only be used for a limited range of viscosities. In our opinion, a parameter that better reveals the applicability of the viscosity sensor is its dynamic range, which is equal to the ratio of fluorescence lifetimes at infinite and zero viscosity at room temperature (τ_η=*∞*_/τ_η=0_). As shown by simulated time-resolved fluorescence decays in Figure S6 (ESI), if the ratio is not sufficiently high, the sensor shows similar response at both high and low viscosities. Therefore, its applicability suffers and a high constant *x* would not make this sensor a useful viscosity probe. Since our goal is to determine the values of the energy barrier that are suitable for a viscosity sensor, we derived how τ_η=*∞*_/τ_η=0_ depends on the energy barrier:

(4) $$\frac{\tau_{\eta =\infty }}{\tau_{\eta =0}}=\tau_{max}\cdot \left(\frac{1}{\tau_{min}}\cdot e^{-\frac{E_{a}}{kT}}+\frac{1}{\tau_{max}}\right)=\frac{\tau_{max}}{\tau_{min}}\cdot e^{-\frac{E_{a}}{kT}}+1.$$

Full derivation of Equation (4) is provided in the ESI. Equation (4) shows that the dynamic range of the probe depends on two parameters: the ratio τ_max_/τ_min_ and the height of the energy barrier (*E*_a_). The lifetimes τ_min_ and τ_max_ correspond to zero viscosity, infinite temperature, and infinite viscosity, 0 K temperature, respectively.

**Figure 7:**
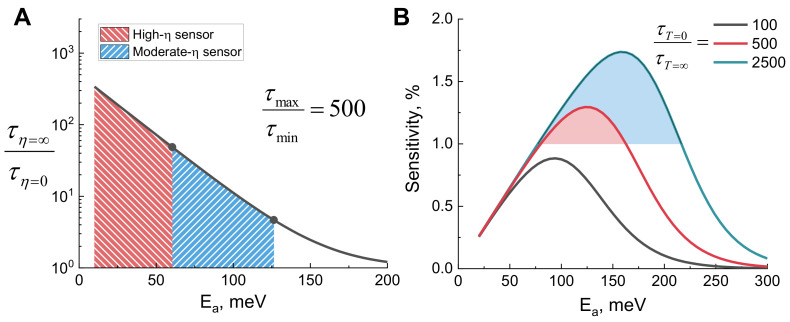
(**A**) Dynamic range of a viscosity sensor, expressed as a ratio of fluorescence lifetimes at zero and infinite viscosities, with respect to the energy barrier height for non-radiative viscosity-dependent relaxation. τ_max_/τ_min_ was set to 500 and T was set to 298 K. The shaded areas correspond to energy barrier values that would be appropriate for a moderate-viscosity sensor and a sensor suitable for high viscosities only. (**B**) Sensitivity to the temperature of the fluorescent temperature sensor with respect to the height of the energy barrier for non-radiative relaxation, which is temperature-dependent. The curves were calculated for three different τ_T=0_/τ_T=∞_ ratios: 100 (black), 500 (red), and 2500 (blue). The shaded areas correspond to energy barrier values resulting in sensitivity above 1%.

The τ_max_/τ_min_ ratio for the derivatives examined in this research can be obtained from the fitting parameters in Table S1, ESI, and is approximately equal to 500. Figure 7A displays how the dynamic range of viscosity probe depends on the *E*_a_ when the τ_max_/τ_min_ ratio is equal to this value. The blue colored region shows dynamic range values between 5 and 50 and is considered a good dynamic range for a moderate-viscosity sensor. The upper bound (τ_η=*∞*_/τη=0>50, the red colored area) is set by the typical time resolution of TCSPC or FLIM [48]. A viscosity sensor with τ_η=*∞*_/τη=0>50 could only be used for imaging high-viscosity environments, as its fluorescence lifetime at moderate viscosities would be too fast for the usual TCSPC or FLIM setups.

The calculations show that acceptable values of *E*_a_ for a viscosity sensor with similar molecular structure to our investigated compounds (Figure 1) are 0.05–0.12 eV, preferably closer to 0.05 eV. These values depend slightly on τ_max_ and τ_min_, which are set by the radiative decay constant and the degree of geometrical change occurring during non-radiative relaxation, respectively. The dependencies when the ratio τ_max_/τ_min_ equals 100 and 2500 are shown in Figure S7 (ESI).

Next, we proceeded to theoretically estimate the optimal energy barrier height *E*_a_ for a fluorescent temperature sensor. The key parameter for a temperature sensor is its temperature sensitivity, which has the following expression:

(5) $$s=-\frac{\partial \tau /\partial T}{\tau }\cdot 100\%\;,$$

where *s* is sensitivity, *T* is temperature, and τ is fluorescence lifetime. Starting with Equation (S1) (ESI), the following dependence of sensitivity on *E*_a_ can be obtained:

(6) $$s=\frac{E_{a}}{T^{2}k\left(1+\frac{\tau_{T=\infty }}{\tau_{T=0}}e^{\frac{E_{a}}{kT}}\right)}\cdot 100\%\;,$$

where τ_T=∞_ and τ_T=0_ are fluorescence lifetimes at infinite and 0 K temperature, respectively, *k* is Boltzmann’s constant, and *T* is temperature. The full derivation of Equation (6) can be found in the ESI. Using this equation, we calculated how sensitivity to temperature depends on the energy barrier for non-radiative relaxation (Figure 7B) for three different τ_T=0_ ⁄ τ_T=∞_ ratios. The results show that the optimal values of *E*_a_ (0.10–0.20 eV) are slightly higher than those for a viscosity probe. The ratio τ_T=0_ ⁄ τ_T=∞_ is also important, as, for instance, if it is equal to 100, it will not be possible to reach a sensitivity of 1% at any *E*_a_ value. As the value of the ratio increases (500 or 2500), it becomes easier to develop a temperature probe with good sensitivity. To get a high ratio, a molecule needs to be able to relax fast at high temperatures, thus giving a low τ_T=∞_ value. This would be the case if the molecular geometry needs to change as little as possible during temperature-dependent non-radiative relaxation. Furthermore, our calculations show that it may be very challenging to obtain a fluorescent temperature probe with sensitivity exceeding 2%. Fluorescent temperature sensors based on a completely different mechanism may be required to reach sensitivities higher than that, as in the work of Xue et al. [49] and Pietsch et al. [50].

**Figure 8:**
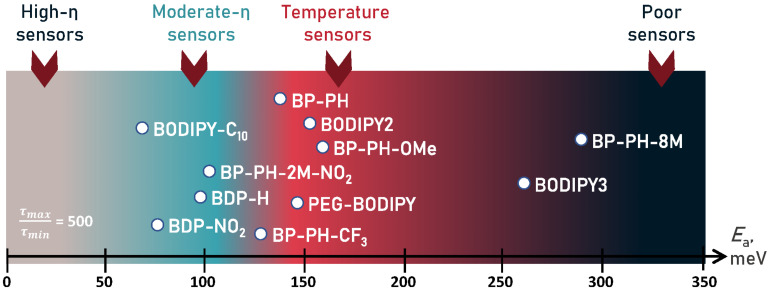
Visual scale demonstrating which energy barrier values are required for different types of sensors. From left: light grey—high-viscosity sensors, sky blue—moderate-viscosity sensors, red—temperature sensors, dark grey—poor sensors. White dots show energy barrier values of BODIPY derivatives reported in this paper (BP-PH-CF_3_, BP-PH-OMe, BP-PH-8M), together with the barriers for other derivatives reported by Toliautas et al. (BDP-H, BDP-NO_2_) [41], Polita et al. (BODIPY-C_10_) [47], Ogle et al. (PEG-BODIPY) [9], Maleckaite et al. (BP-PH, BP-PH-2M-NO_2_) [33], and Vysniauskas et al. (BODIPY2, BODIPY3) [10].

In Figure 8, we show guidelines for energy barrier values required to obtain a fluorescent viscosity sensor or a temperature sensor. We also show $E_{a}$ values of BODIPY molecules investigated in this research, together with some previously reported $E_{a}$ values of BODIPY probes [9,10,33,41]. The scale represents which kind of sensor a BODIPY-based fluorophore is likely to be, depending on the value of the activation energy barrier, when the ratio τ_max_ ⁄ τ_min_ is equal to 500, which is an approximate value for BODIPY probes investigated in this work. Two alternative scales for the ratios of 100 and 2500 are shown in Figure S8, ESI. The results show that viscosity probe requires a relatively small energy barrier of < 120 meV and ideally below 100 meV. Otherwise, the probe may also have substantial sensitivity to temperature, which is not generally desired. The most popular BODIPY viscosity probe BODIPY-C_10_ satisfies this condition. The optimal barrier height for a temperature probe is above 120 meV, where the viscosity-sensitivity is unlikely to be strong. This is where probes BP-PH, BP-PH-CF_3_, and BP-PH-OMe are located, although the temperature sensitivity of the latter is overshadowed by the strong polarity sensitivity. If the energy barrier exceeds 200 meV, as is the case with BP-PH-8M, the fluorophore is unlikely to show strong sensitivity to either viscosity or temperature. Knowing these guidelines makes it possible to estimate viscosity or temperature sensitivities of new probes before synthesis by calculating energy barrier values using DFT.

## 3. Materials and Methods

### 3.1. Dyes, Reagents, and Solvents

BODIPY-C_10_ and BP-PH were synthesised as previously reported [21]. The synthesis of previously unreported derivatives, BP-PH-CF_3_, BP-PH-OMe, and BP-PH-8M, was accomplished using Suzuki reaction and is described in the ESI. Reagents and solvents for the organic synthesis of the BODIPY molecules were purchased directly from commercial suppliers; solvents were purified by known procedures. Thin layer chromatography was performed using TLC-aluminum sheets with silica gel (Merck 60 F254). Visualization was accomplished by UV light. Column chromatography was performed using silica gel 60 (0.040–0.063 mm) (Merck). NMR spectra were recorded on a Bruker Ascend 400 spectrometer (400 MHz for ^1^H, 100 MHz for ^13^C, 128.4 MHz for ^11^B, 376.5 MHz for ^19^F). NMR spectra were referenced to residual solvent peaks. Melting points were determined in open capillaries with a digital melting point IA9100 series apparatus (Thermo Fischer Scientific) and were not corrected. Stock solutions for all dyes were prepared in toluene at a concentration of 2 mM and diluted for further experiments in solvents or their mixtures. Cyclohexane, toluene, castor oil, chloroform, dichloromethane (DCM), dichloroethane (DCE), dimethyl sulfoxide (DMSO), and methanol were obtained from Sigma–Aldrich. The viscosities of toluene/castor oil mixtures were measured by using a vibrational viscometer (SV10, A&D) at temperatures of interest.

### 3.2. Absorption, Steady-State, Time-Resolved Fluorescence, and Quantum Yields

Absorption spectra were measured by using a Jasco V-670 spectrophotometer. Fluorescence spectra and time-resolved fluorescence were recorded with a Edinburgh-F900 (Edinburgh Instruments) fluorimeter. A WhiteLase Micro (Fianium) laser was used as an excitation source together with bandpass filters (Thorlabs), with 10 nm bandwidth centred at 520 nm (BP-PH-8M) and 570 nm (BP-PH, BP-PH-CF_3_, BP-PH-OMe). Excitation of the previously reported BODIPY-C_10_ was performed by using a picosecond pulsed diode laser EPL-470 (Edinburgh Instruments), emitting at 473 nm at 1 MHz frequency. Fluorescence decays were measured using the time-correlated single-photon counting technique. Fluorescence decays had 5000 counts at the peak of the decay, with 20 ns (BP-PH, BP-PH-CF_3_, BP-PH-OMe, BP-PH-8M) and 50 ns (BODIPY-C_10_) windows being used with 4096 channels. Absorption and fluorescence measurements were performed using quartz cuvettes (10 mm). QY measurements were obtained by a comparative method using fluorescein (QY_F_ = 0.95 in 0.1 M NaOH_aq_; used for BODIPY-C_10_) [51], Rhodamine-6G (QY_F_ = 0.94 in EtOH; used for BP-PH-8M) [52], and Rhodamine-101 (QY_F_ = 0.91 in EtOH; used for BP-PH-CF_3_, BP-PH, BP-PH-OMe) [52] as a standard. The concentration of dyes was 2–6 μM.

### 3.3. Theoretical Calculations

Quantum chemical calculations of the studied molecular rotors were performed using the electronic structure modeling package Gaussian09. [53] The calculations were based on density functional theory (DFT) [54] (for ground state properties) and time-dependent DFT (TD-DFT) [55] (for the excited state properties). M06-2X hybrid functional [56] and cc-pVDZ basis sets [57] were used at all stages of the calculations; this use was previously validated by functional benchmarks by Momeni et al. [58]. The conductor-like polarizable continuum model (C-PCM) [59] with solvent parameters of toluene was used to account for bulk solvent effects on the solute molecules.

### 3.4. Data Analysis

An Edinburgh-F900 software package was used for fitting fluorescence decays. For biexponential fluorescence decays, intensity-weighted lifetimes were calculated (Equation (7)):

(7) $$\bar{\tau }=\frac{\sum_{i}a_{i}\tau_{i}^{2}}{\sum_{i}a_{i}\tau_{i}}\;,$$

where *a* is an amplitude value and τ is the value of the lifetime.

The goodness-of-fit parameter ($\chi^{2})$ was 1.5 or less for single decays. Further data processing and analysis were done with Origin 2018.

## 4. Conclusions

In conclusion, we reported new β-phenyl-substituted BODIPY fluorophores showing red-shifted absorption and emission. We investigated the sensitivity of the molecules to viscosity, temperature, and solvent polarity. While BP-PH-8M did not show significant sensitivity, we showed that BP-PH-CF_3_ is a moderate temperature probe. Furthermore, we showed that BP-PH-OMe has an exceptional combination of attractive properties. The fluorophore is a sensitive lifetime-based polarity sensor, it absorbs and emits in the red region of the visible spectrum, it has minimal sensitivity to other parameters, and it exhibits monoexponential decay kinetics.

Additionally, we analysed photophysical parameters that determine the viscosity or temperature sensitivity. Our theoretical results demonstrate that the sensitivity to viscosity and temperature strongly depends on the energy barrier for non-radiative relaxation. The optimal values of the barrier for a temperature probe are in the range of 100–200 meV, while a microviscosity probe should have a smaller barrier of 120 meV or less. We hope that these guidelines will help to develop new viscosity and temperature sensors as they make it easier to estimate the degree of viscosity or temperature sensitivity of probes before synthesis using theoretically calculated energy barrier values.

## Figures and Tables

**Table 1:** Theoretically calculated and experimental values of the peak maxima of absorption ($\lambda_{A}$) and fluorescence emission ($\lambda_{F}$) spectra, as well as Stokes shifts (${\tilde{\nu }}_{SS}$) for investigated derivatives in toluene. Experimental values of fluorescence lifetime (τ), quantum yield (QY), radiative (*k*_r_), and non-radiative (*k*_nr_) decay rates in toluene are also displayed.

	Theoretical			Experiment						
**Derivative**	**$\lambda_{A}$, nm**	**$\lambda_{F}$, nm**	**${\tilde{\nu }}_{\mathrm{SS}}$, cm${}^{-1}$**	**$\lambda_{A}$, nm**	**$\lambda_{F}$, nm**	**${\tilde{\nu }}_{\mathrm{SS}}$, cm${}^{-1}$**	**τ, ns**	**QY, %**	***k*** **_r_, 10^8^ × s${}^{-1}$**	***k*** **_nr_, 10^8^ × s${}^{-1}$**
BODIPY-C_10_	423	443	1094	500	515	583	0.8	12	1.5	11
BP-PH-8M	465	489	1087	530	565	1169	4.3	87	2	0.3
BP-PH-CF_3_	476	518	1703	575	610	998	4	57	1.5	1
BP-PH	485	535	1959	590	630	1076	3.1	28	0.9	2.3
BP-PH-OMe	510	576	2258	625	680	1294	1.6	12	0.8	5.5

**Table 2:** The relative sensitivities to temperature, polarity, and Förster–Hoffmann constant *x* showing the extent of viscosity sensitivity of BODIPY-C_10_, BP-PH-8M, BP-PH-CF_3_, BP-PH, and BP-PH-OMe.

Derivative	[%/°C]	[%/Δf]	*x*
BODIPY-C_10_	0.9	157	0.21
BP-PH-8M	0.3	72	0.02
BP-PH-CF_3_	0.5	116	0.04
BP-PH	0.6	141	0.05
BP-PH-OMe	0.4	265	0.03

## Data Availability

Raw data available upon reasonable request.

## Supplementary Materials

Supplementary materials are available online. Figure S1: Absorbance and fluorescence spectra, Figure S2: Quantum chemical calculations, Figure S3: HOMO and LUMO orbitals, Figure S4: Temperature sensitivity in toluene, Figure S5: Viscosity-lifetime dependencies in toluene-castor oil mixtures, Figure S6: Simulated time-resolved fluorescence decays for different τ_η=*∞*_/τη=0>50 ratios, Figure S7: τ_η=*∞*_/τη=0>50 dependency on the energy barrier for non-radiative relaxation, Figure S8: Visual scale for BODIPY-based rotors when the ratio τ_max_ ⁄ τ_min_ is equal to 100 (A) and 2500 (B), Figures S9–S24: NMR spectra of derivatives studied in this research, Derivation: Equation (4), Derivation: Equation (6), DFT data: The Cartesian (XYZ) coordinates of the optimized ground and excited states for BP-PH-CF_3_, BP-PH-OMe, and BP-PH-8M, Synthesis: Reaction scheme and procedure. Table S1: Obtained fitting parameters from the fits in Figure S1.
