# Supplementary material for: Give or Take: Effects of Electron-Accepting/-Withdrawing Groups in Red-Fluorescent BODIPY Molecular Rotors
Source: Molecules. 2021 Dec 21;27(1):23. doi: 10.3390/molecules27010023 (PMC8746292; doi:10.3390/molecules27010023)
Supplement: Supplementary file 1 [file molecules-27-00023-s001.zip › molecules-1503012-supplementary.pdf]

SUPPORTING INFORMATION

Give or Take: Effects of Electron-Accepting/-Withdrawing Groups in Red-Fluorescent BODIPY Molecular Rotors

Karolina Maleckaitė[1], Domantas Narkevičius[2], Rugilė Žilėnaitė[2], Jelena Dodonova-Vaitkūnienė[2], Stepas Toliautas[3], Sigitas Tumkevičius[2] and Aurimas Vyšniauskas[1,2]*

[1] *Center of Physical Sciences and Technology, Saulėtekio av. 3, Vilnius, LT-10257, Lithuania. E-mail: aurimas.vysniauskas@ftmc.lt*

[2] *Institute of Chemistry, Faculty of Chemistry and Geosciences, Vilnius University, Naugarduko str. 24, Vilnius, LT-03225, Lithuania*

[3] *Institute of Chemical Physics, Faculty of Physics, Vilnius University, Saulėtekio av. 9-III, Vilnius, LT-10222, Lithuania*

Table of contents

1. Absorbance and fluorescence spectra 3

2. Quantum chemical calculations 4

3. Temperature and viscosity sensitivities 6

4. Estimating a required E_a for temperature or microviscosity probes. Calculations and derivations of expressions. 9

5. The Cartesian (XYZ) coordinates of the optimized ground and excited states for BP-PH-CF_3, BP-PH-OMe, and BP-PH-8M 15

6. Synthesis and NMR spectra of BP-PH-OMe, BP-PH-CF_3 and BP-PH-8M 35

References 45

1. Absorbance and fluorescence spectra

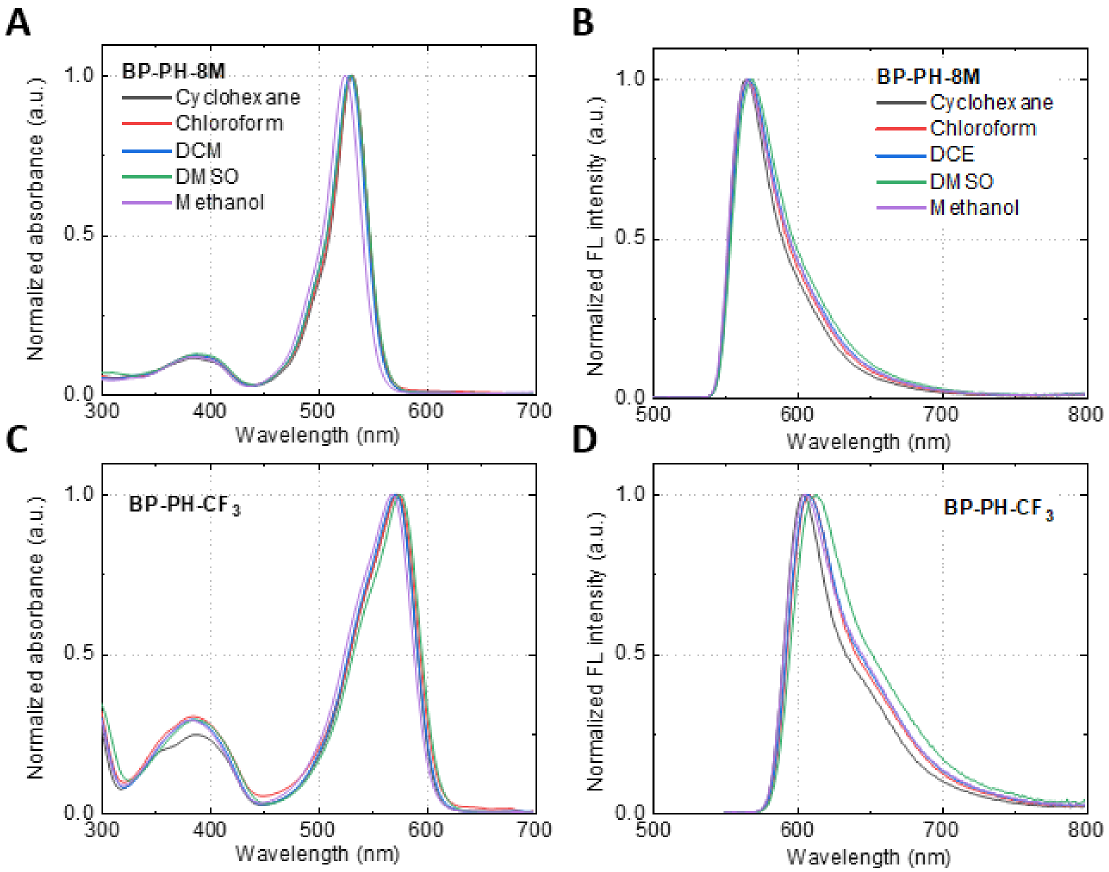

Figure S1. Absorbance (A, C) and fluorescence emission (B, D) spectra of BP-PH-8M and BP-PH-CF_3 in solvents of different polarity. Recorded spectra of BP-PH-8M and BP-PH-CF_3 show very small solvatochromic shifts.

2. Quantum chemical calculations

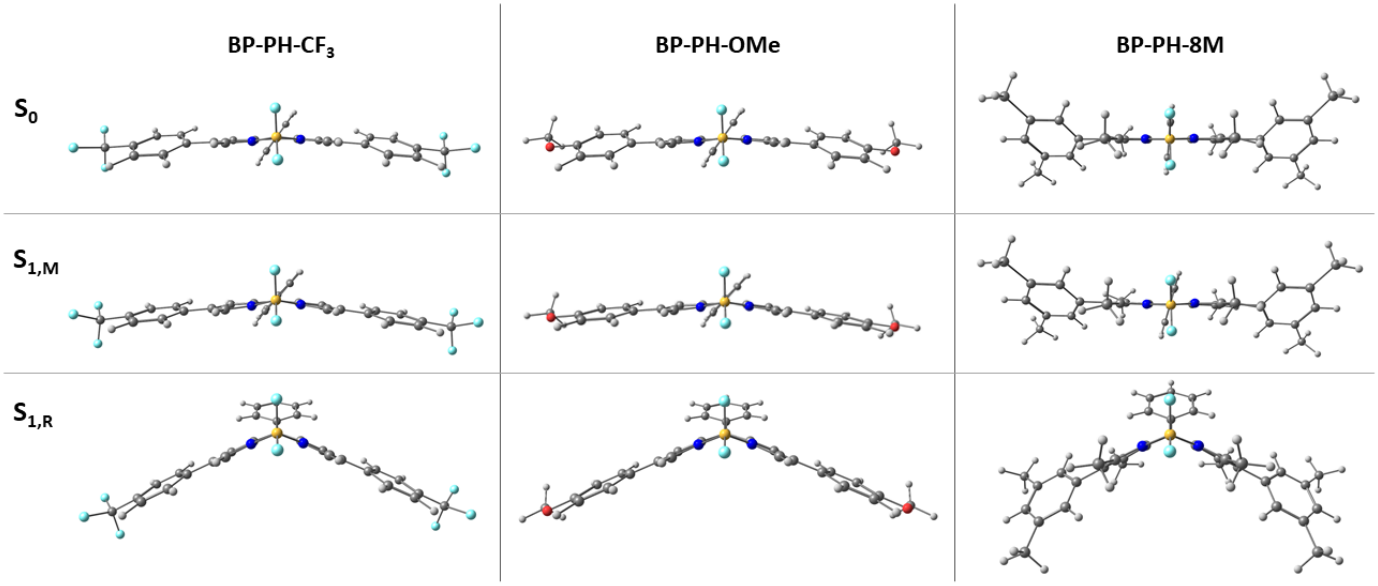

Figure S2. Density functional theory (DFT) optimized structures of BP-PH-CF_3, BP-PH-OMe, BP-PH-8M. Displayed geometries are of the ground state (S_0), as well as the two local minima of the first excited state (S_1) at θ ≈ 45° dihedral angle ($S_{1,m}$), and at θ ≈ 0° ($S_{1,r}$) reachable after crossing an activation energy barrier.

DFT results show that the geometries of trifluoromethyl- and methoxy-substituted molecules are very similar. The dihedral angle θ (between BODIPY core and *meso*-phenyl) is around 45° before (S_0) and right after ($S_{1,m}$) excitation. β-phenyls are planar, which results in an increased molecular conjugation along with red-shifted absorption and fluorescence spectra compared to unsubstituted BODIPY.

BP-PH-8M results are completely the opposite: The dihedral angle θ before excitation (S_0) is around 90° and slightly decreases to 75° after excitation ($S_{1,m}$). β-phenyls are rotated out of conjugation due to the steric hindrance caused by methyl groups. This explains the smaller red-shift of absorption and fluorescence spectra.

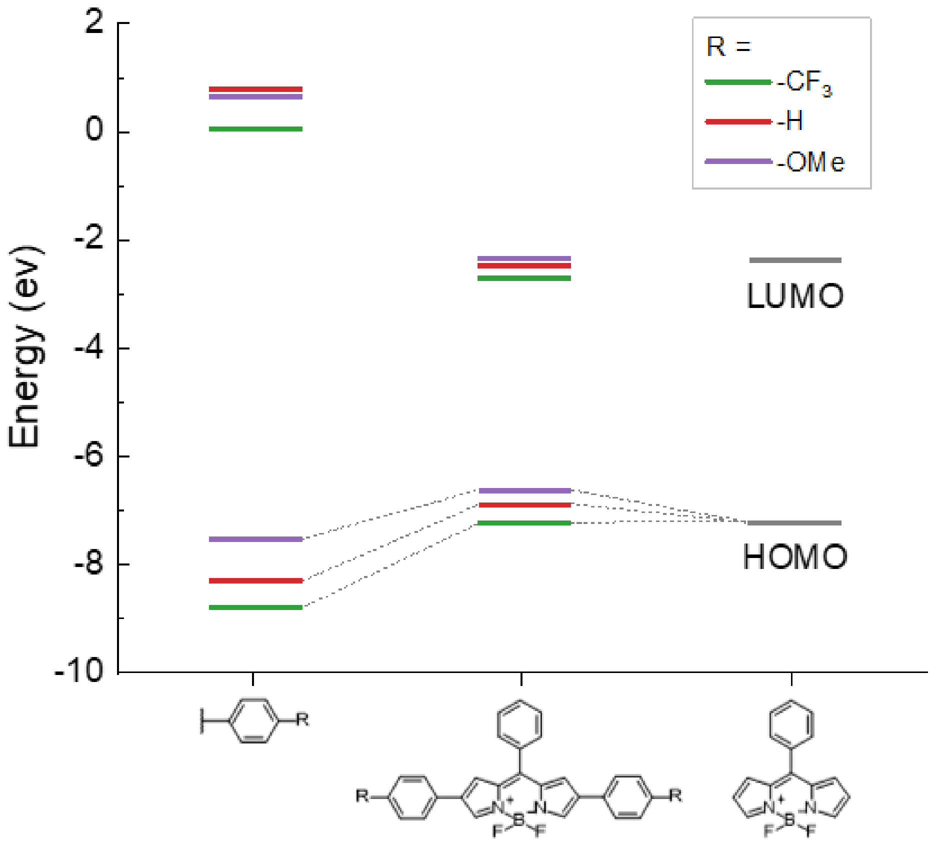

Figure S3. Values of highest occupied molecular orbitals (HOMO) and lowest unoccupied molecular orbitals (LUMO) for individual trifluoromethyl- (green), hydrogen- (red), methoxy- (purple) substituted β-phenyls (on the left), BODIPY together with substituted β-phenyls (in the middle), and pure BODIPY (grey, on the right).

Calculations show that LUMO of a resulting molecule barely depends on LUMO of a substituent due to a significant energy gap. In contrast, a closer energy match between the HOMO of the unsubstituted BODIPY and the β-phenyl substituents results in a higher HOMO level and stronger red-shift of absorption and fluorescence wavelengths, most evident for BP-PH-OMe conjugate.

3. Temperature and viscosity sensitivities

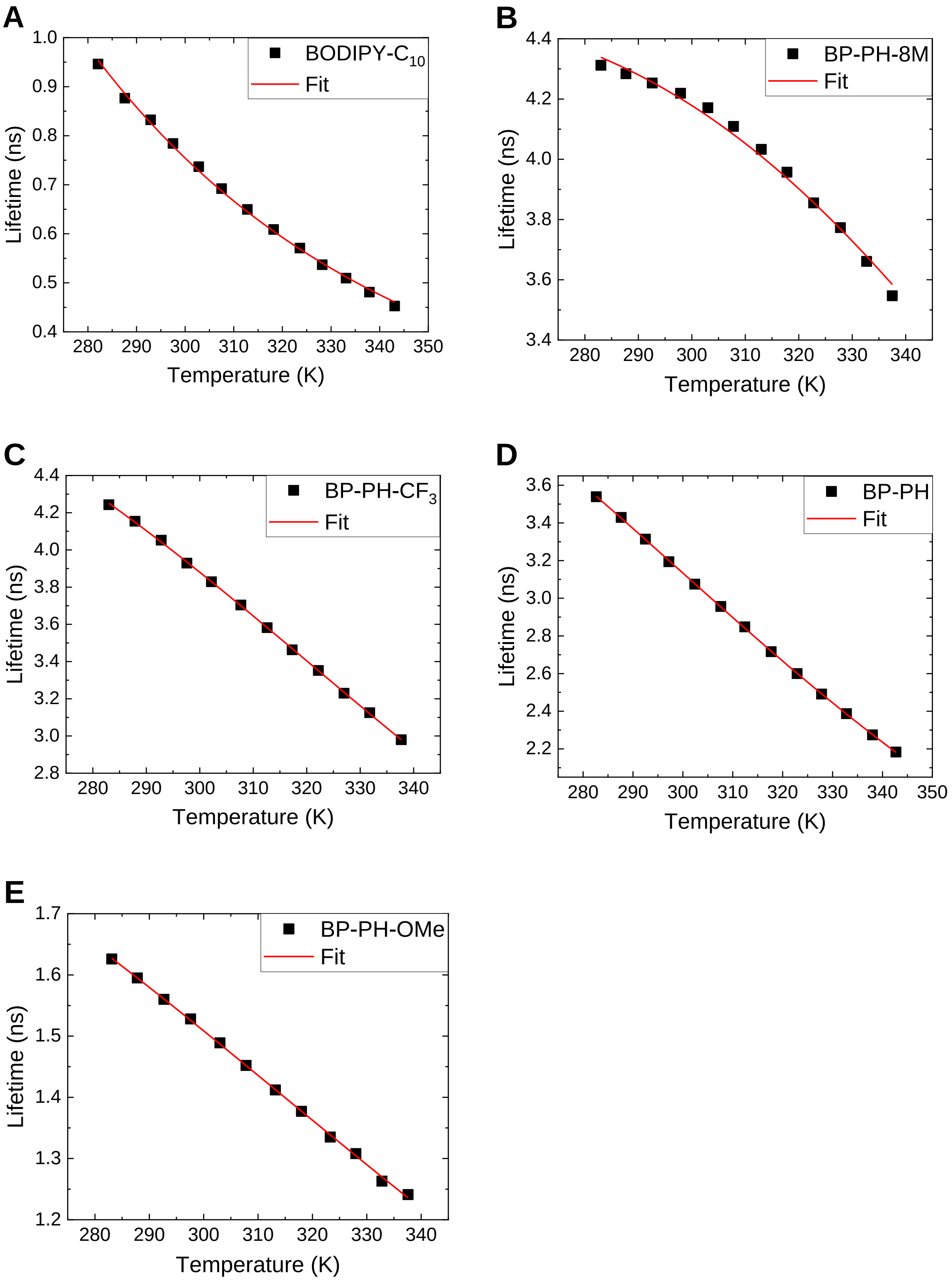

Figure S4. Fits of temperature-dependent lifetimes of BODIPY-C_{10} (A), BP-PH-8M (B), BP-PH-CF_3 (C), BP-PH (D), and BP-PH-OMe (E) in toluene. Equation (S1) was used as a fitting function:

$$\tau = \frac{1}{k_x + k_{nr,max} e^{\left(-\frac{E_a}{kT}\right)}}, \qquad \text{(S1)}$$

where τ is fluorescence lifetime, k_x – the sum of radiative and all temperature-independent non-radiative decay rates leading to the relaxation from the excited state, $k_{nr,max}$ – is a maximum temperature-dependent decay rate, E_a – activation energy barrier, k – Boltzmann's constant, T – temperature. The obtained fitting parameters are displayed in Table S1. The obtained barrier heights match the theoretically calculated ones very well (Figure 3B, main text).

Table S1. Obtained fitting parameters from the fits in Figure S1.

**Derivative**	**k_x (ns^{-1})**	**$k_{nr,max}$ (ns^{-1})**	**E_a (eV)**
BODIPY-C_{10}	0.2	97	0.12
BP-PH	0.2	75	0.17
BP-PH-8M	0.21	411	0.26
BP-PH-CF_3	0.19	75	0.18
BP-PH-OMe	0.49	43	0.14

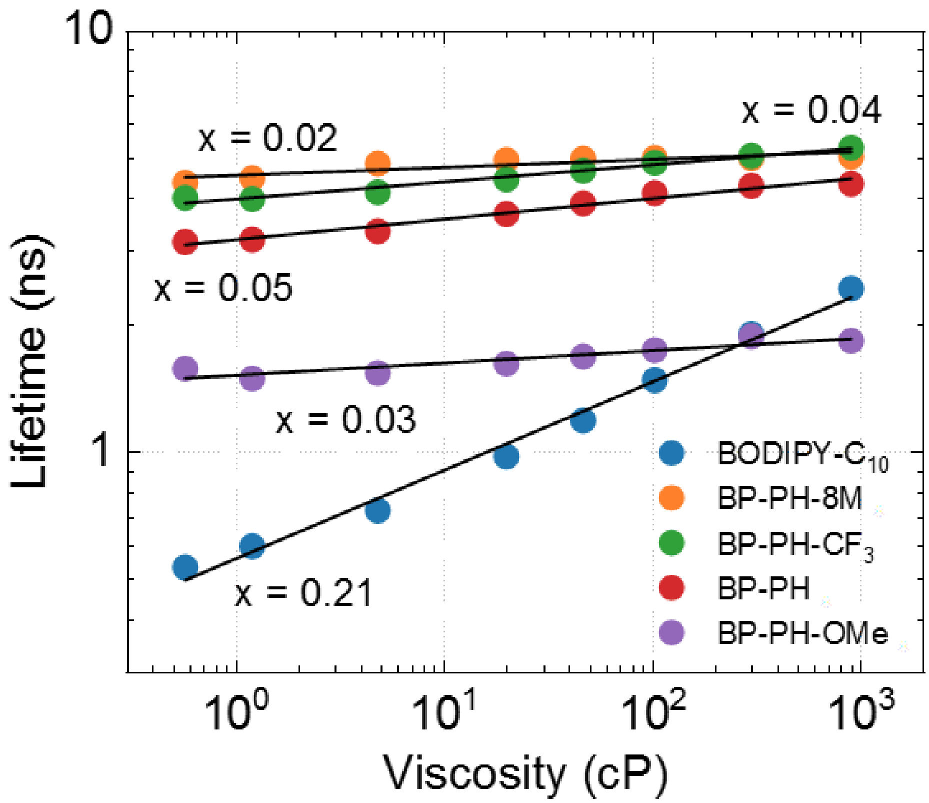

Figure S5. Viscosity-lifetime dependences of BODIPY-C_{10}, BP-PH-8M, BP-PH-CF_3, BP-PH and BP-PH-OMe fitted using Förster-Hoffmann equation [1,2]:

$$\tau = C\eta^{x}, \tag{S2}$$

where τ is fluorescence lifetime, η is viscosity, C and x are constants. The constant x approximately shows the degree of sensitivity to viscosity.

Black lines represent the linear fits with the corresponding constants x written next to them.

4. Estimating a required E_a for temperature or microviscosity probes. Calculations and derivations of expressions.

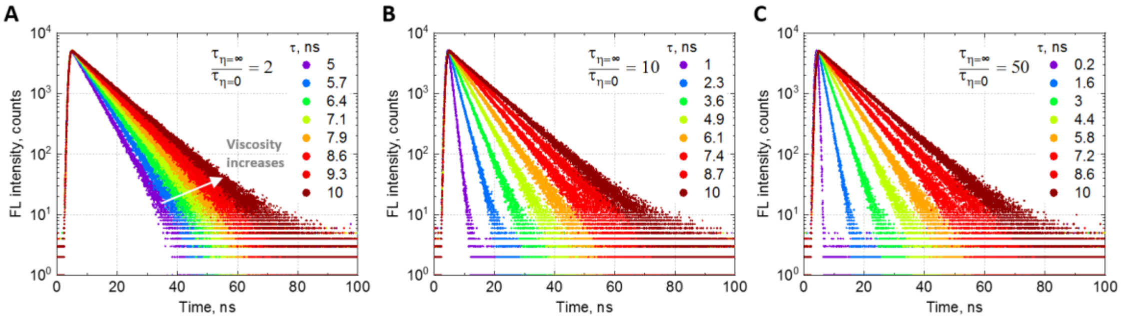

Figure S6. Simulated time-resolved fluorescence decays, when the ratio $\tau_{\eta=\infty}/\tau_{\eta=0}$ is equal to 2 (A), 10 (B), and 50 (C). The fluorescence decays of a viscosity probe would progressively get longer with increasing viscosity (white arrow in Fig.S6A) until the maximum possible lifetime is reached, which was set to 10 ns for the simulation. $\tau_{\eta=\infty}/\tau_{\eta=0}$ effectively determines the dynamic range of the viscosity sensor. If it is too low, the fluorescence response of the probe at the lowest viscosity barely differs from the one at the highest, as in A). In contrast, a high ratio would lead to the large difference in fluorescence response at different viscosities as in C), which is desired for a fluorescent viscosity probe.

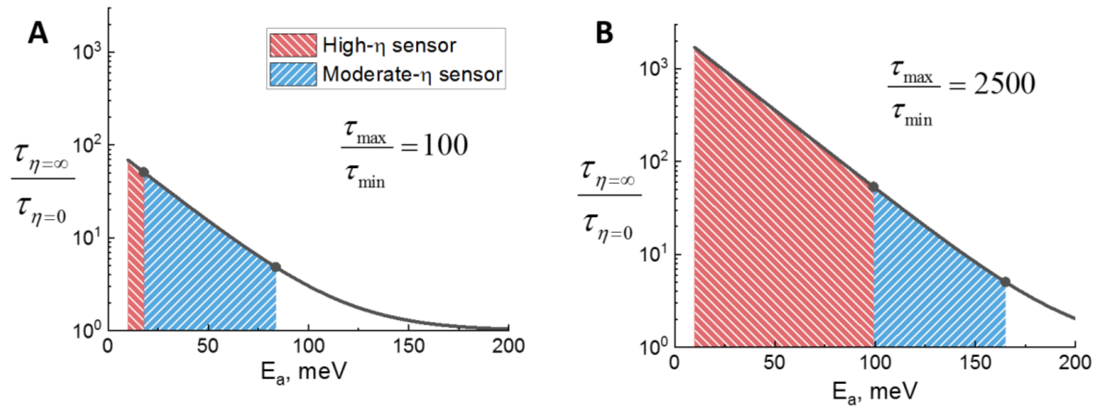

Figure S7. $\tau_{\eta=\infty}/\tau_{\eta=0}$ dependency on the energy barrier for non-radiative relaxation (E_a). Curves for two different τ_{max}/τ_{min} ratios are shown – 100 (A) and 2500 (B). We set $\tau_{\eta=\infty}/\tau_{\eta=0} = 5$ as the minimum sufficient dynamic range for a viscosity sensor. On the other side, the dynamic range when $\tau_{\eta=\infty}/\tau_{\eta=0} > 50$ becomes difficult to exploit, as a typical maximum lifetime for a fluorophore is 10 ns [3], while the time resolution for fluorescence lifetime imaging microscopy (FLIM) setups is usually close to 0.2 ns [4]. If the ratio $\tau_{\eta=\infty}/\tau_{\eta=0}$ is above 50, such viscosity probe would be suitable for high-viscosity environments only, as its fluorescence lifetime at moderate-viscosities will be below the time resolution of typical FLIM setups. Therefore, the area shaded in blue shows a region where the activation energy barrier height is sufficient for a fluorophore to be an applicable viscosity sensor. The area shaded in red shows E_a values that would lead to a viscosity sensor for high-viscosity environments only.

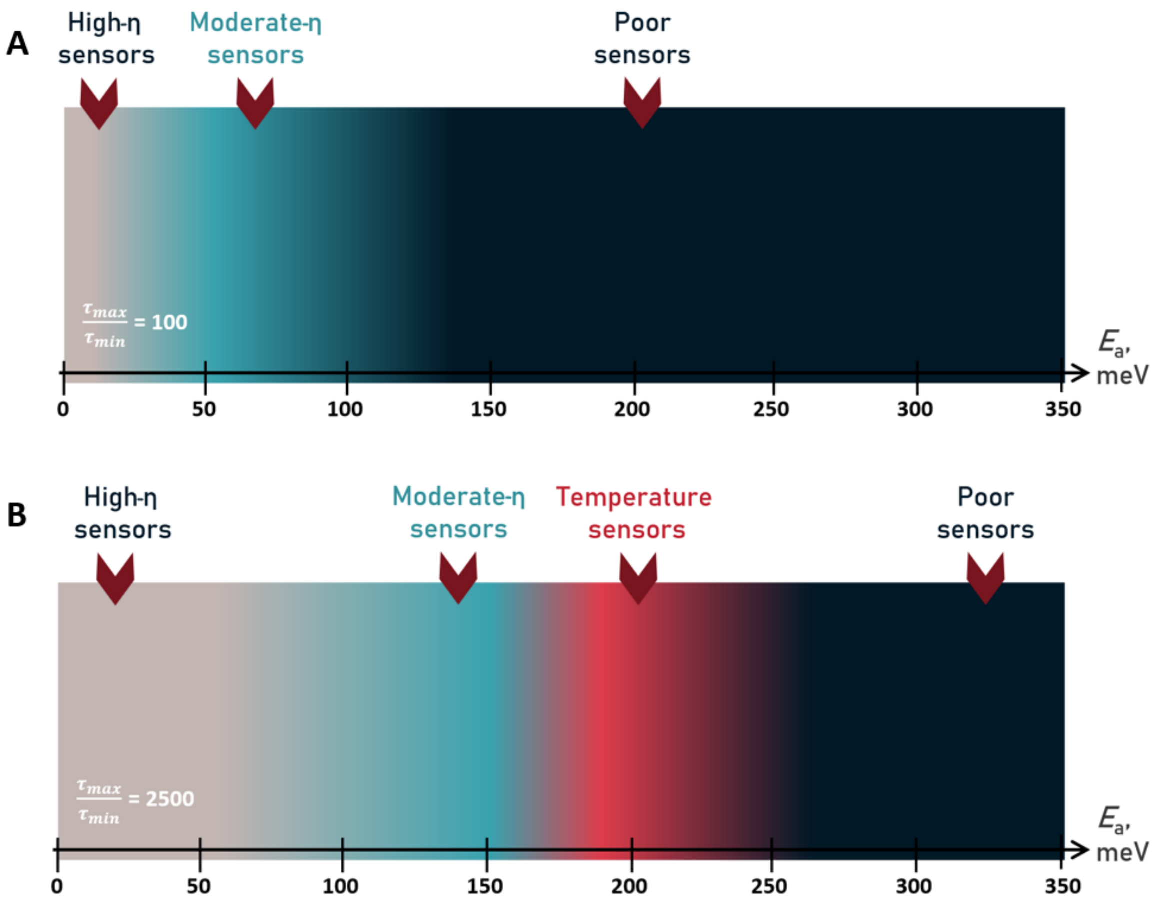

Figure S8. Visual scale for BODIPY-based rotors, their activation energy and possible sensing properties when the ratio τ_{max}/τ_{min} is equal to 100 (A) and 2500 (B). Visualised in colour (from left: light grey – high-viscosity sensors, sky blue – moderate-viscosity sensors, red – temperature sensors, dark grey – poor sensors).

Figure S8 shows that when the τ_{max}/τ_{min} is very low (100), it is not possible to create temperature sensor. This would be the case if there are very significant changes in the molecular geometry during the temperature-dependent non-radiative relaxation. This would make the relaxation slow leading to a large τ_{min}. Moreover, creating a viscosity probe becomes a very hard task because of the limited range of activation energy barrier values. If the ratio is very high (2500), i.e. molecular geometry changes little during relaxation, it becomes easier to create a viscosity probe as well as a temperature sensor.

Derivation of Equation (4)

Equation (S3) shows how fluorescence lifetime depends on viscosity and temperature:

$$\tau = \frac{1}{\frac{1}{C\eta^x + \frac{1}{k_{nr,max}}} \cdot e^{-\frac{E_a}{kT}} + k_r + k_x} = \frac{1}{\frac{1}{C\eta^x + \tau_{min}} \cdot e^{-\frac{E_a}{kT}} + \frac{1}{\tau_{max}}}, \quad \text{(S3)}$$

where τ is a fluorescence lifetime, η – dynamic viscosity, C and x – constants, E_a – activation energy for non-radiative relaxation, $k_{nr,max}$ – non-radiative decay constant at zero viscosity and infinite temperature, k – Boltzmann's constant, T – temperature in Kelvin, k_r – radiative decay constant, k_x – the sum of any other rate viscosity- and temperature-independent constants that lead to the population loss from the fluorescent state, τ_{min} and τ_{max} – minimal and maximum possible fluorescence lifetimes of the probe, respectively.

Thus, the fluorescence lifetimes at room temperature and at zero and infinite viscosity ($\tau_{\eta=0}$ and $\tau_{\eta=\infty}$, respectively) are as follows:

$$\tau_{\eta=0} = \frac{1}{\frac{1}{C0^x + \tau_{min}} \cdot e^{-\frac{E_a}{kT}} + \frac{1}{\tau_{max}}} = \frac{1}{\frac{1}{\tau_{min}} \cdot e^{-\frac{E_a}{kT}} + \frac{1}{\tau_{max}}}, \quad \text{(S4)}$$

$$\tau_{\eta=\infty} = \frac{1}{\frac{1}{C\infty^x + \tau_{min}} \cdot e^{-\frac{E_a}{kT}} + \frac{1}{\tau_{max}}} = \frac{1}{0 + \frac{1}{\tau_{max}}} = \tau_{max}\,. \quad \text{(S5)}$$

Then the ratio $\tau_{\eta=\infty}/\tau_{\eta=0}$, which corresponds to the dynamic range of a viscosity probe, is equal to the following:

$$\frac{\tau_{\eta=\infty}}{\tau_{\eta=0}} = \tau_{max} \cdot \left(\frac{1}{\tau_{min}} \cdot e^{-\frac{E_a}{kT}} + \frac{1}{\tau_{max}}\right) = \frac{\tau_{max}}{\tau_{min}} \cdot e^{-\frac{E_a}{kT}} + 1\,. \quad \text{(S6)}$$

Temperature sensitivity dependence on E_a. Formula derivation.

The applicability of temperature sensor can be evaluated by calculating temperature sensitivity s of the probe. The bigger the change of the lifetime with respect to the temperature change, the more sensitive is the probe. Such sensitivity can be expressed by the following expression:

$$s = -\frac{\partial\tau/\partial T}{\tau} \cdot 100\%, \tag{S7}$$

here $\partial\tau$ – change in fluorescence lifetime, ∂T – change in temperature, τ – fluorescence lifetime.

Starting with Equation (S3), $\frac{\partial\tau}{\partial T}$ can be written as:

$$\frac{\partial\tau}{\partial T} = \frac{\partial\left(\frac{1}{k_{nr,max}e^{-\frac{E_a}{kT}} + k_x}\right)}{\partial T} = -\frac{k_{nr,max}e^{-\frac{E_a}{kT}} \cdot E_a}{T^2 k \cdot \left(k_{nr,max}e^{-\frac{E_a}{kT}} + k_x\right)^2}, \tag{S8}$$

where A is a constant, E_a – activation energy, k – Boltzmann's constant, T – temperature in Kelvin, k_x – the sum of any other rate constants that lead to the population loss from the fluorescent state.

Combining Equations (S7) and (S8) gives:

$$\frac{s}{100\%} = \frac{k_{nr,max}e^{-\frac{E_a}{kT}} \cdot E_a}{T^2k \cdot \left(k_{nr,max}e^{-\frac{E_a}{kT}} + k_x\right)^2} \cdot \left(k_{nr,max}e^{-\frac{E_a}{kT}} + k_x\right)$$

$$= \frac{k_{nr,max}e^{-\frac{E_a}{kT}} \cdot E_a}{T^2k \cdot \left(k_{nr,max}e^{-\frac{E_a}{kT}} + k_x\right) \cdot \frac{e^{-\frac{E_a}{kT}}}{e^{-\frac{E_a}{kT}}}} = \tag{S9}$$

$$= \frac{k_{nr,max} \cdot E_a}{T^2k \cdot \left(k_{nr,max} + k_x \cdot e^{\frac{E_a}{kT}}\right)} = \frac{E_a}{\tau_{T=\infty} \cdot T^2k\left(\frac{1}{\tau_{T=\infty}} + \frac{e^{\frac{E_a}{kT}}}{\tau_{T=0}}\right)}$$

$$= \frac{E_a}{T^2k\left(1 + \frac{\tau_{T=\infty}}{\tau_{T=0}}e^{\frac{E_a}{kT}}\right)}.$$

5. The Cartesian (XYZ) coordinates of the optimized ground and excited states for BP-PH-CF_3, BP-PH-OMe, and BP-PH-8M

BP-PH-CF_3
======
S_0

N	-1.239169	-1.285018	0.124901
C	-2.508369	-1.684974	0.081067
C	-3.378544	-0.566389	0.017786
C	-2.555005	0.559279	0.035924
C	-1.216636	0.103788	0.088741
C	0.731354	3.019869	0.989044
C	0.738417	4.411323	0.935981
C	0.043410	5.077643	-0.073638
C	-0.665424	4.349178	-1.029359
C	-0.688900	2.958047	-0.973172
C	0.011718	2.282641	0.036995
C	0.000431	0.805910	0.098763
C	1.214607	0.100154	0.146595
C	2.550559	0.548602	0.022477
C	3.371499	-0.579248	0.026980
C	2.500542	-1.692565	0.149175
N	1.234612	-1.286297	0.220426
F	0.028305	-3.257735	-0.493327
B	-0.010602	-2.204643	0.407549
F	-0.067904	-2.667331	1.716707
H	-2.753044	-2.744245	0.126754
H	-2.862009	1.600675	0.000572
H	1.262318	2.495789	1.784938
H	1.287687	4.977256	1.688719

H	0.055185	6.167148	-0.116481
H	-1.200541	4.865981	-1.826280
H	-1.226543	2.385789	-1.730361
H	2.852827	1.584443	-0.104633
H	2.740427	-2.752667	0.197976
C	-4.846489	-0.613893	-0.042793
C	-5.612163	0.496885	0.343070
C	-5.510825	-1.768289	-0.483037
C	-6.999952	0.458956	0.285301
C	-6.899607	-1.813335	-0.537216
C	-7.642785	-0.696764	-0.158165
H	-5.115960	1.394499	0.713461
H	-4.937204	-2.639902	-0.800099
H	-7.587277	1.322651	0.597047
H	-7.407123	-2.716331	-0.876063
C	4.836238	-0.631998	-0.084442
C	5.613803	0.496948	0.212878
C	5.486441	-1.808662	-0.487376
C	6.999159	0.456916	0.104432
C	6.871625	-1.856005	-0.590699
C	7.626903	-0.720225	-0.300170
H	5.129970	1.413088	0.553065
H	4.903951	-2.696340	-0.736005
H	7.595448	1.336451	0.346268
H	7.368066	-2.776106	-0.899410
C	-9.139029	-0.721887	-0.260951
F	-9.718753	0.045971	0.674748
F	-9.632405	-1.962099	-0.126821
F	-9.567739	-0.264217	-1.450569
C	9.117754	-0.761876	-0.459802
F	9.621597	-1.965899	-0.147090

F	9.733111	0.145245	0.313682
F	9.493831	-0.507872	-1.725506

--

BP-PH-CF_3

======

$S_{1,m}$

N	-1.225682	-1.232512	0.279668
C	-2.474192	-1.665609	0.143995
C	-3.364446	-0.557457	0.033246
C	-2.561378	0.599212	0.112971
C	-1.227821	0.180008	0.242901
C	0.841295	3.084464	1.168963
C	0.877008	4.475738	1.153582
C	0.099776	5.189406	0.240090
C	-0.711704	4.499661	-0.661231
C	-0.752863	3.108013	-0.647427
C	0.020462	2.377736	0.271926
C	-0.006867	0.907635	0.296308
C	1.211300	0.171090	0.291396
C	2.536446	0.590199	0.098873
C	3.343127	-0.565632	0.046410
C	2.459307	-1.672599	0.216120
N	1.213770	-1.237684	0.368819
F	0.007502	-3.282908	-0.078519
B	-0.019409	-2.119348	0.679213
F	-0.074455	-2.417858	2.039170
H	-2.689534	-2.731253	0.171480
H	-2.890702	1.631344	0.068186
H	1.434581	2.530615	1.898431
H	1.510084	5.006348	1.865593
H	0.128753	6.279318	0.228849

H	-1.309998	5.048619	-1.389268
H	-1.362394	2.575474	-1.378569
H	2.846037	1.620125	-0.045232
H	2.674491	-2.738029	0.248133
C	-4.808515	-0.640269	-0.109385
C	-5.601741	0.521914	-0.039524
C	-5.449735	-1.878239	-0.317630
C	-6.980996	0.451284	-0.174657
C	-6.828143	-1.950808	-0.452724
C	-7.593736	-0.784718	-0.385690
H	-5.131866	1.489223	0.136043
H	-4.862604	-2.794083	-0.380610
H	-7.586961	1.354649	-0.109454
H	-7.314929	-2.913502	-0.607905
C	4.781552	-0.649359	-0.142532
C	5.568224	0.519609	-0.163463
C	5.424462	-1.893036	-0.305496
C	6.942047	0.449286	-0.342895
C	6.798125	-1.965272	-0.484829
C	7.556528	-0.793275	-0.508002
H	5.097001	1.492108	-0.023897
H	4.842947	-2.814489	-0.296313
H	7.543426	1.358173	-0.347755
H	7.286434	-2.932150	-0.604140
C	-9.080257	-0.861737	-0.578598
F	-9.718907	0.145223	0.034716
F	-9.586633	-2.010043	-0.105082
F	-9.417548	-0.799585	-1.878035
C	9.036507	-0.866553	-0.747410
F	9.560957	-2.014604	-0.294340
F	9.690335	0.140535	-0.149740

F	9.333325	-0.796084	-2.056179

BP-PH-CF_3

======

$S_{1,r}$

N	-1.204371	0.045535	1.575525
C	-2.343680	-0.628459	1.486913
C	-3.155146	-0.063919	0.460752
C	-2.412654	1.004992	-0.080980
C	-1.200522	1.078806	0.618847
C	1.209901	3.931571	-0.264541
C	1.206003	5.242760	-0.720317
C	0.002410	5.905662	-0.970034
C	-1.202044	5.243909	-0.721442
C	-1.207628	3.932723	-0.265673
C	0.000697	3.223817	-0.056075
C	-0.000106	1.858718	0.419049
C	1.199269	1.077420	0.618800
C	2.411702	1.002575	-0.080651
C	3.152203	-0.068081	0.460103
C	2.339737	-0.632316	1.485563
N	1.201456	0.043450	1.574658
F	-0.002332	-1.534161	2.949815
B	-0.001327	-0.217598	2.521225
F	-0.000203	0.686216	3.572672
H	-2.540992	-1.459946	2.158829
H	-2.681212	1.616489	-0.935769
H	2.161597	3.459088	-0.031923
H	2.154952	5.758875	-0.869280
H	0.003063	6.934847	-1.329135
H	-2.150361	5.760909	-0.871353

H	-2.160010	3.461146	-0.034034
H	2.681341	1.614003	-0.935185
H	2.536331	-1.464079	2.157370
C	-4.484614	-0.518971	0.077814
C	-5.262573	0.224643	-0.829827
C	-5.021315	-1.710976	0.600688
C	-6.525746	-0.209185	-1.206176
C	-6.284652	-2.147112	0.226012
C	-7.034712	-1.398409	-0.681731
H	-4.877990	1.160898	-1.233606
H	-4.441449	-2.309445	1.302979
H	-7.123955	0.378306	-1.902505
H	-6.692250	-3.069347	0.639340
C	4.481277	-0.524703	0.077436
C	5.266081	0.226013	-0.819547
C	5.010362	-1.724312	0.588429
C	6.528191	-0.209005	-1.194785
C	6.273914	-2.162652	0.213619
C	7.030581	-1.406838	-0.680896
H	4.886642	1.168165	-1.214441
H	4.424301	-2.328945	1.280262
H	7.131681	0.383767	-1.882625
H	6.674537	-3.092311	0.616142
C	-8.380807	-1.890568	-1.127277
F	-9.214956	-0.878612	-1.410067
F	-8.970804	-2.652415	-0.194859
F	-8.293859	-2.639319	-2.239951
C	8.381870	-1.882156	-1.128669
F	8.886834	-2.816413	-0.311560
F	9.267686	-0.875214	-1.187290
F	8.336404	-2.421307	-2.358908

BP-PH-OMe

======

S_0

N	-1.240098	-1.236652	0.130528
C	-2.511694	-1.634916	0.094937
C	-3.383060	-0.517488	0.027534
C	-2.557183	0.606387	0.035117
C	-1.217132	0.151278	0.088037
C	0.730055	3.070340	0.975458
C	0.738321	4.461702	0.915801
C	0.045514	5.123926	-0.097973
C	-0.661777	4.390791	-1.051175
C	-0.685114	2.999739	-0.988505
C	0.012808	2.328256	0.025782
C	0.001020	0.850619	0.093647
C	1.215364	0.146364	0.143511
C	2.553843	0.592657	0.021406
C	3.375544	-0.534257	0.034163
C	2.501995	-1.645306	0.158374
N	1.233961	-1.239227	0.221934
F	0.025726	-3.204719	-0.505795
B	-0.011304	-2.156111	0.403148
F	-0.066151	-2.634183	1.708808
H	-2.757857	-2.693647	0.146360
H	-2.864849	1.647446	-0.006700
H	1.260632	2.549338	1.773614
H	1.287039	5.030792	1.666683
H	0.057766	6.213273	-0.145962
H	-1.196022	4.903786	-1.851250
H	-1.222948	2.423666	-1.742649

H	2.858664	1.627505	-0.108503
H	2.742679	-2.705071	0.212353
C	-4.851625	-0.569165	-0.024304
C	-5.626580	0.520335	0.383360
C	-5.522443	-1.714992	-0.486491
C	-7.020340	0.487823	0.335658
C	-6.906184	-1.764754	-0.534852
C	-7.668902	-0.662114	-0.125074
H	-5.136677	1.417028	0.766598
H	-4.950330	-2.578284	-0.830540
H	-7.583884	1.357860	0.667555
H	-7.429105	-2.650236	-0.896332
C	4.841239	-0.592731	-0.066573
C	5.630550	0.511027	0.268685
C	5.495186	-1.757534	-0.505204
C	7.021761	0.474822	0.172352
C	6.876233	-1.811188	-0.600809
C	7.653301	-0.693837	-0.264041
H	5.154282	1.423152	0.632236
H	4.911593	-2.633535	-0.793126
H	7.596770	1.356942	0.447943
H	7.385892	-2.711502	-0.944161
O	-9.014762	-0.805377	-0.211100
C	-9.811262	0.291085	0.191682
H	-10.851483	-0.017562	0.044938
H	-9.648124	0.535122	1.253681
H	-9.603579	1.182657	-0.421500
O	8.995213	-0.842742	-0.392149
C	9.805923	0.266631	-0.059315
H	10.840258	-0.049705	-0.229409
H	9.578360	1.134433	-0.698978

H	9.679253	0.551690	0.997458

--

BP-PH-OMe

======

$S_{1,m}$

N	-1.223081	-1.201802	0.300414
C	-2.477496	-1.636049	0.172537
C	-3.366451	-0.532431	0.087803
C	-2.557723	0.626333	0.175860
C	-1.225779	0.207332	0.285922
C	0.850358	3.107708	1.227837
C	0.883107	4.499172	1.223638
C	0.092961	5.220035	0.326839
C	-0.728266	4.534441	-0.568989
C	-0.765151	3.142615	-0.566593
C	0.021062	2.403230	0.335652
C	-0.003695	0.933476	0.346427
C	1.216479	0.198631	0.342058
C	2.540799	0.617640	0.172067
C	3.352421	-0.541639	0.111226
C	2.468332	-1.644487	0.252453
N	1.216943	-1.207816	0.395690
F	0.013854	-3.235029	-0.137718
B	-0.016554	-2.096766	0.664553
F	-0.074074	-2.462416	2.012282
H	-2.689622	-2.702344	0.184112
H	-2.889517	1.658447	0.152765
H	1.453787	2.549439	1.945292
H	1.524135	5.024756	1.932566
H	0.119212	6.310147	0.324440
H	-1.337780	5.087426	-1.284864

H	-1.383247	2.614631	-1.293647
H	2.855275	1.648738	0.048299
H	2.679456	-2.710808	0.268467
C	-4.808220	-0.609719	-0.042973
C	-5.604524	0.548301	0.018810
C	-5.467154	-1.846044	-0.237729
C	-6.987604	0.494751	-0.104384
C	-6.839549	-1.913903	-0.362228
C	-7.617437	-0.743260	-0.296711
H	-5.131995	1.518206	0.175152
H	-4.886952	-2.766909	-0.300519
H	-7.564047	1.416045	-0.047338
H	-7.350238	-2.864469	-0.514913
C	4.788567	-0.619852	-0.063901
C	5.578809	0.544086	-0.091649
C	5.449124	-1.861869	-0.214365
C	6.956840	0.491011	-0.260340
C	6.816635	-1.929304	-0.382421
C	7.588173	-0.752619	-0.407699
H	5.104767	1.518295	0.029188
H	4.873815	-2.787792	-0.205721
H	7.528618	1.416846	-0.273569
H	7.328617	-2.884067	-0.500716
O	-8.948020	-0.912579	-0.428776
C	-9.769350	0.240223	-0.366126
H	-10.797194	-0.113828	-0.492701
H	-9.670498	0.744353	0.607771
H	-9.521163	0.947638	-1.172607
O	8.914143	-0.922110	-0.576509
C	9.729228	0.236593	-0.608831
H	10.754227	-0.119123	-0.752420

H	9.447096	0.895793	-1.444421
H	9.661815	0.794331	0.338131

BP-PH-OMe

======

$S_{1,r}$

N	-1.202122	-0.552437	1.334520
C	-2.353036	-1.195311	1.151436
C	-3.178333	-0.453546	0.266337
C	-2.433868	0.699078	-0.084165
C	-1.207498	0.636748	0.584052
C	1.204025	3.593803	0.160308
C	1.198883	4.961698	-0.078128
C	-0.003897	5.656892	-0.219361
C	-1.205860	4.960105	-0.078832
C	-1.209360	3.592228	0.159628
C	-0.002213	2.855360	0.249131
C	-0.001295	1.427950	0.496855
C	1.206311	0.638530	0.583910
C	2.432075	0.702395	-0.084927
C	3.178608	-0.448959	0.265889
C	2.354809	-1.191554	1.151717
N	1.203066	-0.550263	1.335036
F	0.001922	-2.365974	2.377332
B	0.000660	-0.988335	2.205634
F	-0.000222	-0.312988	3.421379
H	-2.540929	-2.133729	1.666342
H	-2.717066	1.453079	-0.810353
H	2.156814	3.095334	0.319366
H	2.148379	5.494908	-0.139436

H	-0.004562	6.730589	-0.407590
H	-2.156012	5.492074	-0.140761
H	-2.161594	3.092534	0.318149
H	2.713863	1.456686	-0.811367
H	2.544166	-2.129615	1.666731
C	-4.513830	-0.822617	-0.166575
C	-5.286456	0.044226	-0.958629
C	-5.084966	-2.065985	0.188244
C	-6.564040	-0.296786	-1.387717
C	-6.352334	-2.417751	-0.230452
C	-7.107546	-1.536876	-1.025045
H	-4.883851	1.016690	-1.243018
H	-4.518108	-2.770457	0.797397
H	-7.126703	0.406862	-1.998115
H	-6.793982	-3.376970	0.038989
C	4.514461	-0.815965	-0.167337
C	5.284290	0.050710	-0.962369
C	5.088962	-2.057073	0.190142
C	6.562259	-0.288318	-1.391777
C	6.356755	-2.406841	-0.228834
C	7.109111	-1.526174	-1.026409
H	4.879036	1.021401	-1.249020
H	4.524441	-2.761274	0.801765
H	7.122625	0.415081	-2.004568
H	6.801011	-3.364264	0.042694
O	-8.333023	-1.970468	-1.384446
C	-9.125222	-1.113584	-2.187438
H	-10.063957	-1.647655	-2.364819
H	-9.333656	-0.164862	-1.668785
H	-8.632494	-0.904811	-3.149836
O	8.335250	-1.957715	-1.385812

C	9.124628	-1.101097	-2.191896
H	10.064470	-1.633398	-2.368714
H	8.630460	-0.896147	-3.154372
H	9.331255	-0.150486	-1.676002

BP-PH-8M
======
S_0

N	12.350217	-9.357713	-1.196527
C	13.036552	-9.692582	-2.294717
C	14.005751	-8.689313	-2.568639
C	13.887495	-7.711792	-1.575389
C	12.837371	-8.146924	-0.708533
C	12.199460	-5.075924	0.592875
C	12.699070	-3.867548	1.078213
C	13.808232	-3.858011	1.923983
C	14.421394	-5.059640	2.281962
C	13.927756	-6.268347	1.794135
C	12.813970	-6.278862	0.949037
C	12.277201	-7.571207	0.439450
C	11.215593	-8.188252	1.115359
C	10.492786	-7.818202	2.291404
C	9.545957	-8.826288	2.502809
C	9.706570	-9.784115	1.464766
N	10.694411	-9.394265	0.651262
F	11.572671	-11.455526	-0.268197
B	11.163208	-10.163199	-0.610254
F	10.120818	-10.232804	-1.546602
H	11.329031	-5.091238	-0.065643
H	12.217772	-2.930509	0.795978
H	14.195938	-2.912676	2.305110

H	15.288338	-5.056161	2.943547
H	14.402266	-7.212216	2.069340
C	14.940863	-8.708895	-3.713621
C	15.005548	-7.623430	-4.602373
C	15.764564	-9.814859	-3.939759
C	15.877888	-7.632016	-5.689622
C	16.645825	-9.853041	-5.028081
C	16.691279	-8.756267	-5.888287
H	14.347204	-6.765839	-4.444557
H	15.727764	-10.659745	-3.246973
H	17.376446	-8.774289	-6.740322
C	8.549180	-8.911647	3.589721
C	7.708953	-7.823246	3.876526
C	8.420549	-10.079168	4.347552
C	6.764562	-7.890234	4.899431
C	7.475456	-10.176469	5.376552
C	6.660271	-9.075840	5.639891
H	7.789464	-6.914885	3.274907
H	9.080555	-10.926089	4.142535
H	5.920631	-9.139265	6.442743
C	14.739977	-6.485984	-1.452767
H	14.136769	-5.566924	-1.483583
H	15.286909	-6.474808	-0.500291
H	15.470441	-6.453413	-2.269813
C	10.701050	-6.625437	3.174161
H	11.764295	-6.459419	3.388711
H	10.321485	-5.705311	2.704032
H	10.168208	-6.767632	4.122746
C	12.740605	-10.938563	-3.058199
H	13.210527	-10.901821	-4.047572
H	13.119220	-11.817150	-2.514823

H	11.654866	-11.061492	-3.163726
C	8.936956	-11.038214	1.222603
H	9.522838	-11.914088	1.539330
H	7.994832	-11.021835	1.782573
H	8.732641	-11.148728	0.149977
C	15.947836	-6.467945	-6.644086
H	15.238234	-5.679086	-6.363429
H	16.958020	-6.032462	-6.656991
H	15.717512	-6.788195	-7.670853
C	17.524506	-11.057523	-5.247781
H	18.184210	-11.224106	-4.383461
H	16.918555	-11.966639	-5.375188
H	18.152261	-10.935795	-6.139796
C	5.864459	-6.721877	5.208980
H	6.030283	-6.361150	6.235033
H	6.043690	-5.886776	4.519663
H	4.805947	-7.010578	5.131839
C	7.354638	-11.448818	6.175119
H	7.041891	-12.285509	5.532774
H	8.320553	-11.724825	6.622598
H	6.618524	-11.344450	6.982531

--

BP-PH-8M

======

$S_{1,m}$

N	12.391989	-9.361091	-1.152642
C	13.098523	-9.727959	-2.232282
C	14.053214	-8.706552	-2.533535
C	13.901169	-7.685644	-1.581806
C	12.857947	-8.114325	-0.700174
C	12.008229	-5.030408	0.714157

C	12.452322	-3.794097	1.181410
C	13.661820	-3.699816	1.870656
C	14.426957	-4.846437	2.089182
C	13.985363	-6.081535	1.618220
C	12.772150	-6.184705	0.925728
C	12.284750	-7.501540	0.442990
C	11.211588	-8.147205	1.112400
C	10.495599	-7.801714	2.298571
C	9.552179	-8.825355	2.499431
C	9.711371	-9.775969	1.440722
N	10.693583	-9.362492	0.628679
F	11.549197	-11.445333	-0.254152
B	11.179648	-10.139685	-0.609579
F	10.162802	-10.204576	-1.580038
H	11.061127	-5.109097	0.176745
H	11.851376	-2.900780	1.006444
H	14.007884	-2.733230	2.238646
H	15.370811	-4.778979	2.631685
H	14.577064	-6.982059	1.794415
C	14.972392	-8.746513	-3.680860
C	15.052892	-7.661577	-4.573179
C	15.772132	-9.871798	-3.914977
C	15.911173	-7.692127	-5.669646
C	16.644714	-9.926613	-5.008083
C	16.702396	-8.832330	-5.871825
H	14.409387	-6.793343	-4.415356
H	15.728680	-10.713243	-3.218862
H	17.379332	-8.865268	-6.729927
C	8.562233	-8.928126	3.576295
C	7.820380	-7.798862	3.973383
C	8.335989	-10.145786	4.232962

C	6.874640	-7.878418	4.991749
C	7.394555	-10.250718	5.262302
C	6.674151	-9.112548	5.627645
H	7.973642	-6.851200	3.453175
H	8.925464	-11.022850	3.955926
H	5.935000	-9.183966	6.430225
C	14.723110	-6.436894	-1.486229
H	14.095713	-5.535112	-1.429345
H	15.356233	-6.437712	-0.586775
H	15.381908	-6.350294	-2.358171
C	10.746733	-6.650329	3.224789
H	11.820012	-6.449874	3.337426
H	10.283629	-5.716376	2.867462
H	10.328195	-6.874675	4.214976
C	12.798032	-10.983359	-2.968874
H	13.338453	-11.016157	-3.920823
H	13.072585	-11.860249	-2.360784
H	11.714990	-11.054177	-3.149916
C	8.940971	-11.015770	1.157401
H	9.532814	-11.906850	1.423699
H	8.001466	-11.027264	1.720764
H	8.733813	-11.082626	0.079870
C	15.990675	-6.535262	-6.631813
H	15.307068	-5.727375	-6.341272
H	17.011194	-6.126115	-6.667456
H	15.731412	-6.855406	-7.651691
C	17.503417	-11.144912	-5.230049
H	18.182873	-11.304654	-4.379834
H	16.884093	-12.048589	-5.327041
H	18.110025	-11.044609	-6.139138
C	6.071563	-6.673510	5.408312

H	6.234226	-6.444036	6.471757
H	6.345979	-5.788542	4.820148
H	4.994986	-6.855122	5.274422
C	7.177959	-11.570044	5.957638
H	6.834919	-12.335655	5.246198
H	8.114221	-11.935424	6.404284
H	6.428490	-11.482254	6.754546

BP-PH-8M

======

$S_{1,r}$

N	2.224634	-0.685021	-0.405778
C	2.714948	-1.385793	0.625471
C	2.635948	-0.584007	1.808201
C	2.071691	0.650250	1.442730
C	1.849536	0.584309	0.044644
C	0.846652	3.716768	-1.932822
C	1.139891	5.069088	-2.035372
C	2.092869	5.655202	-1.197133
C	2.778058	4.856366	-0.276988
C	2.491838	3.503202	-0.167272
C	1.490971	2.901793	-0.969638
C	1.212120	1.493772	-0.879040
C	0.241495	0.790560	-1.686261
C	-1.117460	1.057866	-1.985642
C	-1.579768	-0.044664	-2.724785
C	-0.496756	-0.974058	-2.830186
N	0.568237	-0.471618	-2.191113
F	1.815545	-2.526464	-1.920025
B	1.940788	-1.141133	-1.870571

F	2.929921	-0.681299	-2.733017
H	0.138163	3.264545	-2.625160
H	0.630140	5.672563	-2.787342
H	2.319165	6.718429	-1.279285
H	3.555009	5.292726	0.351826
H	3.068243	2.883855	0.518190
C	3.054645	-0.998078	3.153691
C	3.722855	-0.094830	4.000531
C	2.781309	-2.287017	3.629398
C	4.116424	-0.463941	5.284330
C	3.165168	-2.682579	4.915675
C	3.830171	-1.763176	5.727412
H	3.959029	0.905651	3.633066
H	2.233695	-2.989250	2.996694
H	4.134513	-2.061933	6.734203
C	-2.927459	-0.232103	-3.277558
C	-3.605345	0.844591	-3.878200
C	-3.573170	-1.472911	-3.201086
C	-4.889130	0.692054	-4.395515
C	-4.863395	-1.652351	-3.712393
C	-5.505089	-0.564457	-4.305119
H	-3.103567	1.810496	-3.962041
H	-3.073734	-2.310190	-2.708283
H	-6.513395	-0.693965	-4.707693
C	1.628675	1.751211	2.356382
H	2.432984	2.464042	2.594377
H	0.810509	2.325285	1.898832
H	1.277480	1.327776	3.308188
C	-1.927606	2.204550	-1.464183
H	-1.470762	2.615355	-0.552661
H	-2.017628	3.031997	-2.184520

H	-2.946702	1.865362	-1.229743
C	3.298635	-2.747066	0.461816
H	3.964388	-2.975057	1.302464
H	2.507170	-3.511516	0.418904
H	3.856607	-2.813960	-0.480845
C	-0.449787	-2.267309	-3.568847
H	-0.573190	-3.117865	-2.880277
H	-1.249440	-2.302106	-4.318104
H	0.523690	-2.393835	-4.059156
C	4.842327	0.500481	6.186258
H	4.976834	1.475910	5.701525
H	4.286024	0.654992	7.122506
H	5.835126	0.111686	6.456817
C	2.853841	-4.074278	5.403000
H	1.772950	-4.272476	5.358363
H	3.350439	-4.828982	4.775375
H	3.186897	-4.217557	6.438866
C	-5.610601	1.843368	-5.046993
H	-6.552655	2.061482	-4.522581
H	-4.996583	2.753030	-5.042158
H	-5.865288	1.606009	-6.090399
C	-5.536205	-2.997090	-3.611183
H	-4.962966	-3.763504	-4.153495
H	-5.605877	-3.322354	-2.562834
H	-6.550322	-2.967830	-4.029750

---

6. Synthesis and NMR spectra of BP-PH-OMe, BP-PH-CF_3 and BP-PH-8M

Reaction scheme

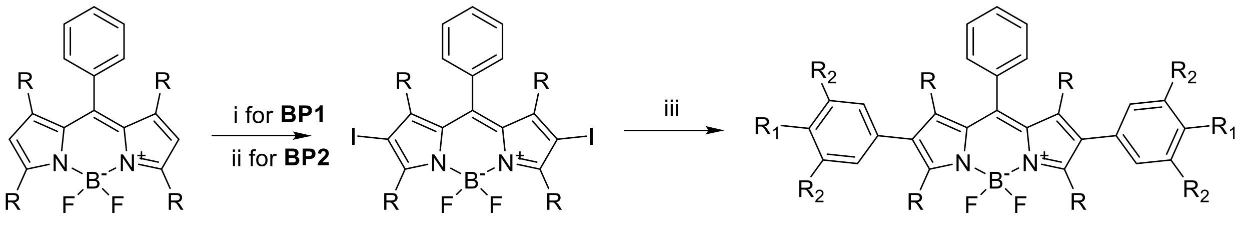

BP1 R=H,
BP2 R=CH_3

BP3 R=H,
BP4 R=CH_3

BP-PH-OMe R=R_2=H, R_1=OCH_3,
BP-PH-CF_3 R=R_2=H, R_1=CF_3,
BP-PH-8Me R=CH_3, R_1=H, R_2=CH_3

Reagents and conditions: i – NIS, CH_2Cl_2; ii –ICl, MeOH/CH_2Cl_2; iii - arylboronic acid, $Pd(OAc)_2$, 2-biPhPCy$_2$, K_3PO_4, toluene, argon, 60 °C, 24h.

Compound **BP1** [5] was synthesized as previously reported. Compounds **BP2** [6] and **BP4** [7] were synthesized using known procedures.

BP3

A methanolic solution of ICl (1.25 mmol, 5 eq.) was added to **BP1** (67.0 mg, 0.25 mmol) previously dissolved in 10 mL CH_2Cl_2 and 10 mL MeOH. The reaction mixture was refluxed (65 °C) and followed by TLC monitoring until complete consumption of the starting material. A saturated solution of sodium thiosulfate was added, followed by extractions with CH_2Cl_2. The organic layer was washed with H_2O and brine and dried over Na_2SO_4. Purification on silica column chromatography using a gradient of toluene/petroleum ether (30/70 to 50/50), followed by precipitation in CH_2Cl_2/EtOH under reduced pressure afforded the bis iodinated **BP3** as a green metallic powder (110.0 mg, 87%), mp 249-250 °C. ^1H NMR ($CDCl_3$): δ (ppm) = 7.92 (s, 2H), 7.66-7.52 (m, 5H), 7.13 (s, 2H). ^{13}C NMR ($CDCl_3$): δ = 148.52, 137.69, 136.05, 133.00, 131.50, 130.43, 130.35, 128.84, 128.83. ^{11}B NMR ($CDCl_3$): δ = -0.31 (t, *J* =28.2 Hz). ^{19}F NMR ($CDCl_3$): δ = -144.68 (q, *J* = 30.1 Hz).

General procedure for the synthesis of compounds BP-PH-OMe, BP-PH-CF_3 and BP-PH-8Me.

To a solution of **BP3** (0.115 mmol) or **BP4** (0.052 mmol), corresponding arylboronic acid (2,4 eq.), K_3PO_4 (4.8 eq.), 2-biPhPCy$_2$ (10 mol%) in toluene (2 mL) Pd(OAc)$_2$ (5 mol%) was added under an argon atmosphere. The mixture was heated at 60 °C for 24 hours, and then cooled to room temperature. Water (10 mL) was added and the mixture was extracted with $CHCl_3$ (2×20 mL), the combined organic layers were dried over Na_2SO_4 and concentrated under reduced pressure. Residue was purified by column chromatography using $CHCl_3$-petroleum ether (2:1) as an eluent.

BP-PH-OMe. Green crystals, yield 82%, mp 225-226 °C. ^{1}H NMR ($CDCl_3$): δ (ppm) = 8.03 (s, 2H), 7.46-7.39 (m, 5H), 7.26 (d, *J* = 8 Hz, 4H), 6.80 (s, 2H), 6.72 (d, *J* = 8 Hz, 4H), 3.63 (s, 6H). ^{13}C NMR ($CDCl_3$): δ = 159.26, 145.65, 141.72, 135.80, 134.09, 133.99, 130.70, 130.50, 128.59, 126.67, 125.30, 124.45, 114.39, 55.36. ^{11}B NMR ($CDCl_3$): δ = 0.22 (t, *J* =28.2 Hz). ^{19}F NMR ($CDCl_3$): δ = -145.25 (q, *J* = 30.1 Hz).

BP-PH-CF_3. Violet crystals, yield 98%, mp 315-316 °C. ^{1}H NMR ($CDCl_3$): δ (ppm) = 8.37 (s, 2H), 7.72-7.63 (m, 13H), 7.21 (s, 2H). ^{13}C NMR ($CDCl_3$): δ = 158.20, 147.74, 142.35, 136.01, 135.98, 131.30, 130.48, 128.86, 126.61, 126.03, 125.99, 125.61, 117.70, 113.13. ^{11}B NMR ($CDCl_3$): δ = 0.21 (t, *J* =28.2 Hz). ^{19}F NMR ($CDCl_3$): δ = -62.52 (s), -144.99 (q, *J* = 26.3 Hz).

BP-PH-8Me. Bright rose crystals, yield 95%, mp 274-275 °C. ^{1}H NMR ($CDCl_3$): δ (ppm) = 7.52-7.51 (m, 3H), 7.40-7.38 (m, 2H), 6.97 (s, 2H), 6.81 (s, 4H), 2.57 (s, 6H), 2.35 (s, 12H), 1.34 (s, 6H). ^{13}C NMR ($CDCl_3$): δ = 154.22, 141.83, 139.06, 138.10, 137.73, 135.56, 133.57, 129.17, 128.94, 128.70, 128.11, 127.92, 125.11, 21.32, 13.42, 12.79. ^{11}B NMR ($CDCl_3$): δ = 1.01 (t, *J* =32.1 Hz). ^{19}F NMR ($CDCl_3$): δ = -146.1 (q, *J* = 32.8 Hz).

NMR spectra

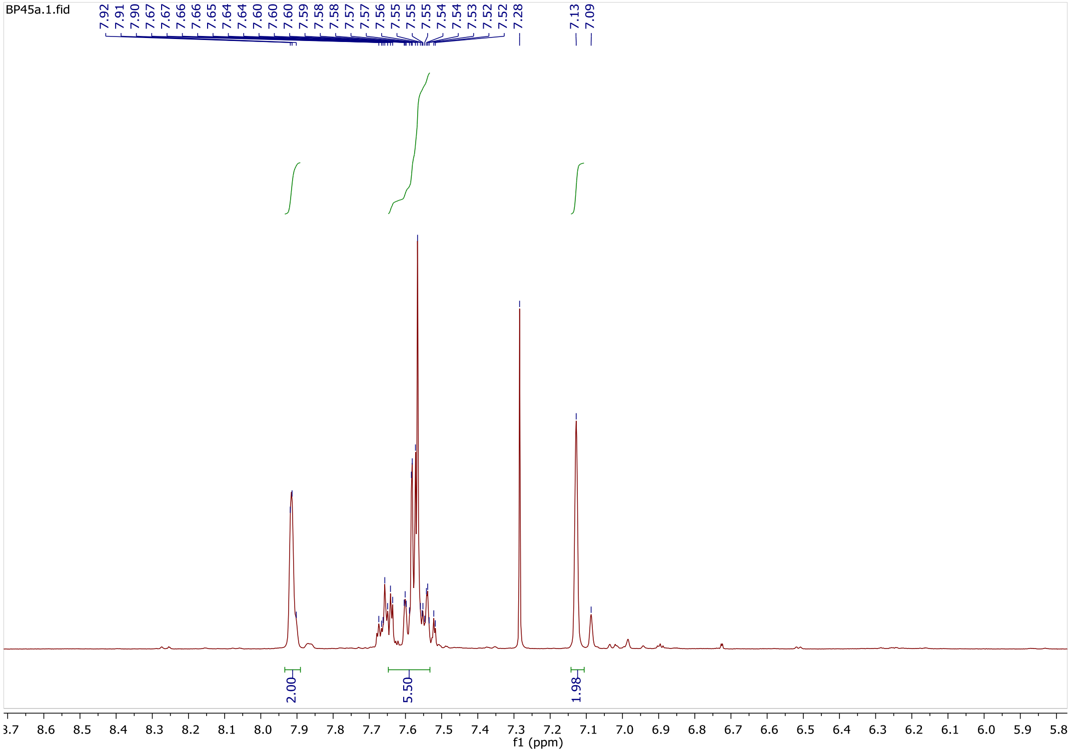

Figure S9. ^{1}H NMR spectrum of **BP3**

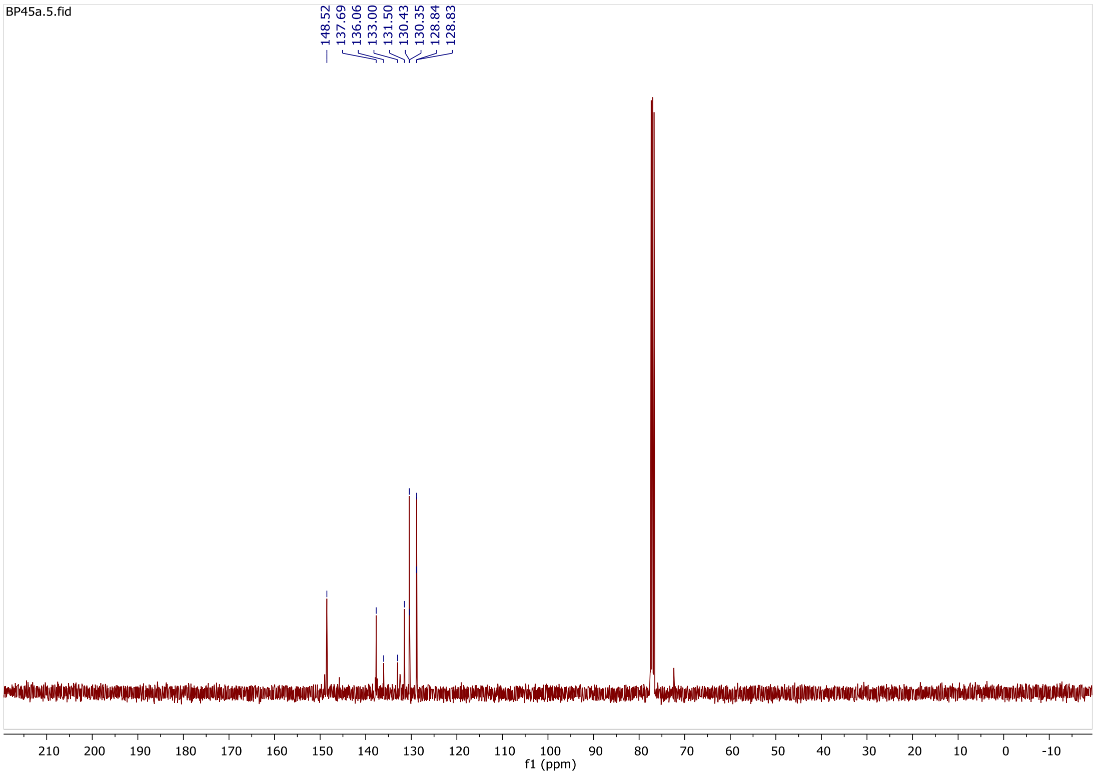

Figure S10. ^{13}C NMR spectrum of **BP3**.

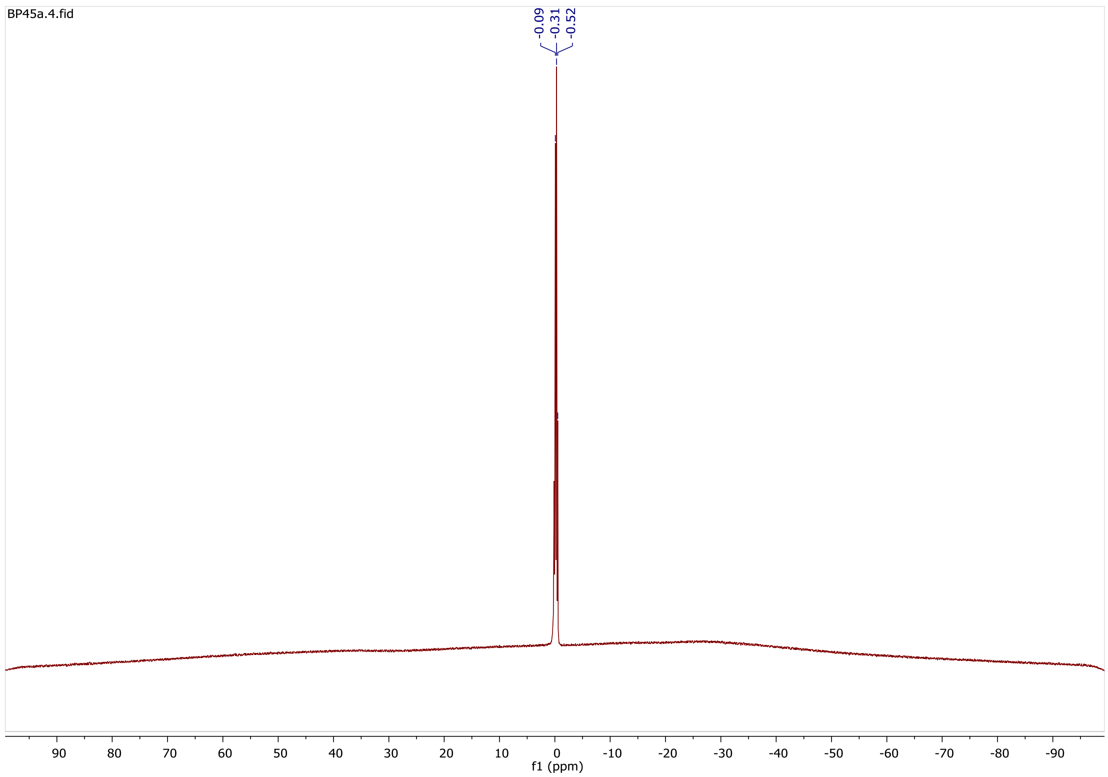

Figure S11. ^{11}B NMR spectrum of **BP3**.

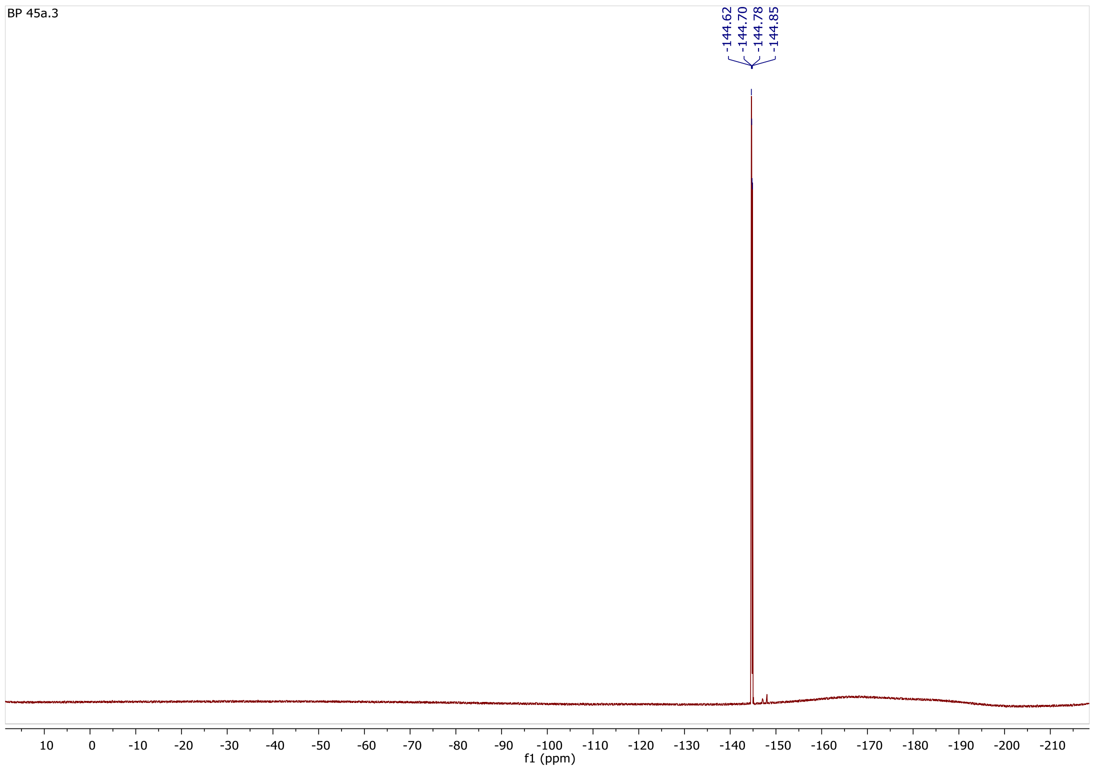

Figure S12. ^{19}F NMR spectrum of **BP3**.

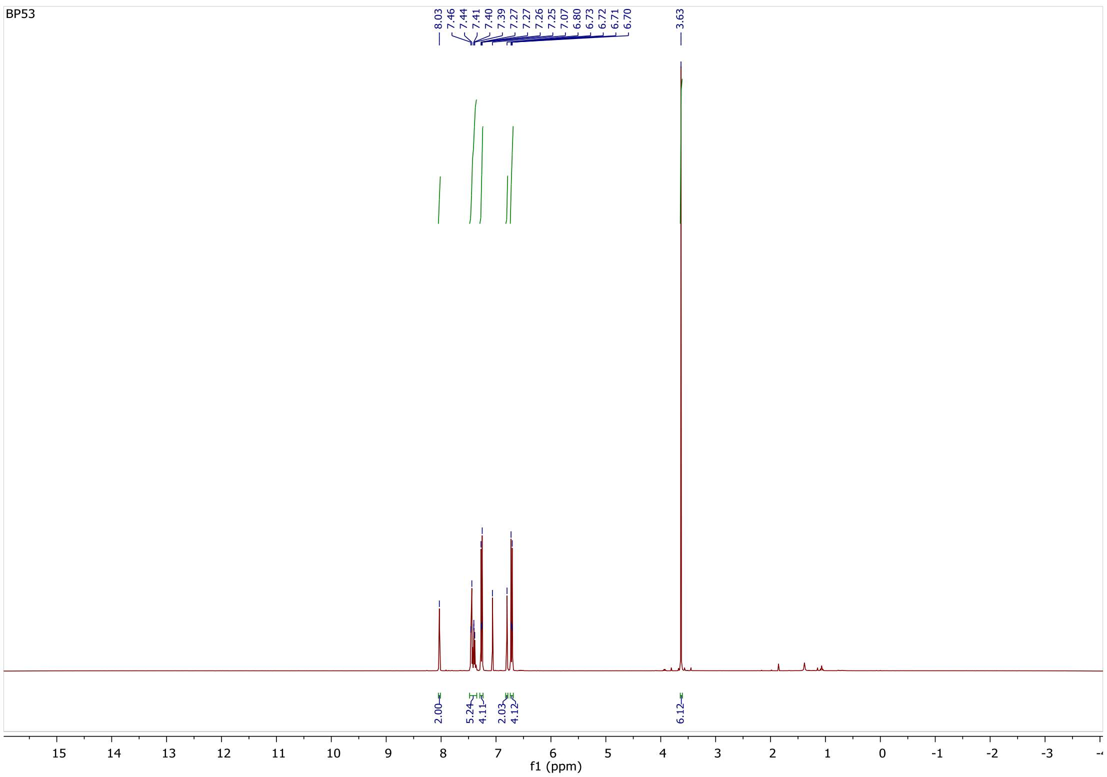

Figure S13. ^{1}H NMR spectrum of **BP-PH-OMe**.

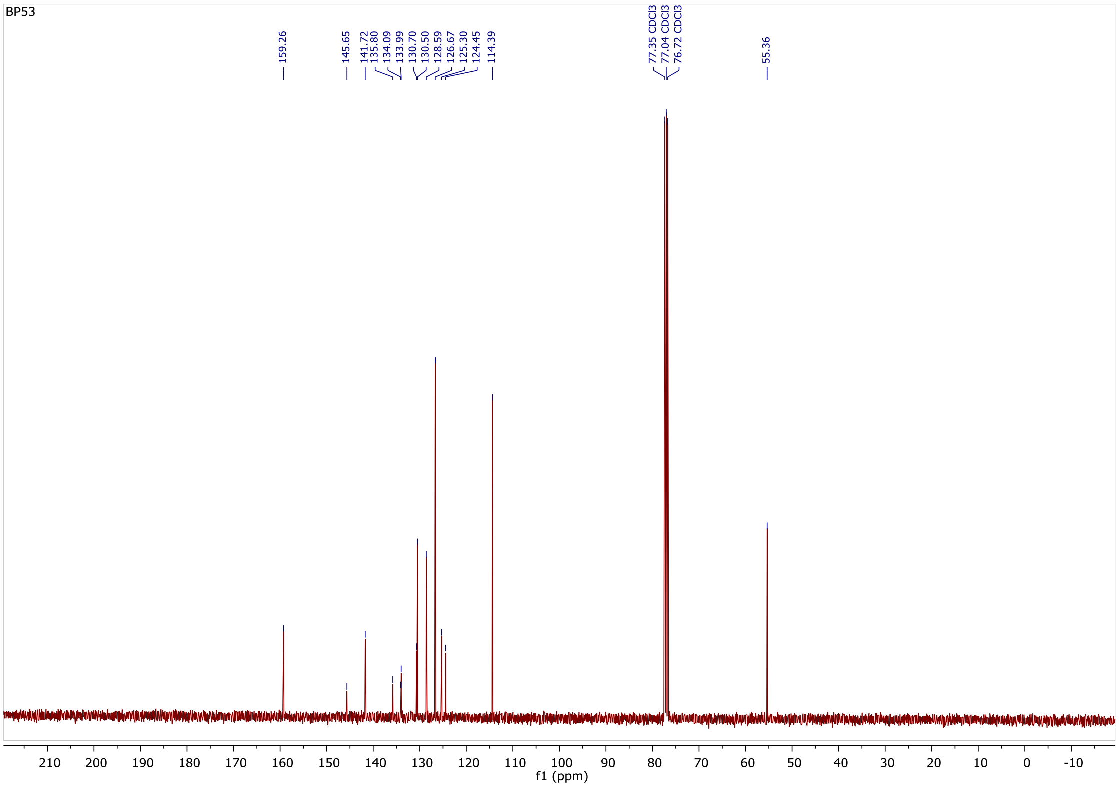

Figure S14. ^{13}C NMR spectrum of **BP-PH-OMe**.

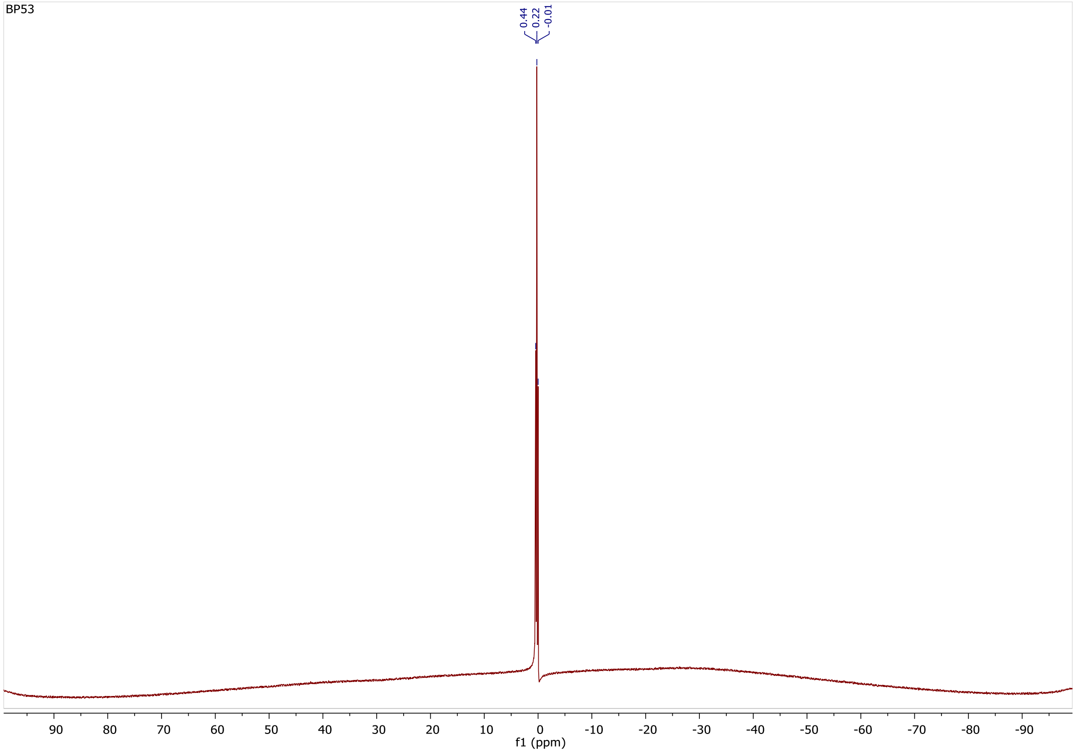

Figure S15. ^{11}B NMR spectrum of **BP-PH-OMe**.

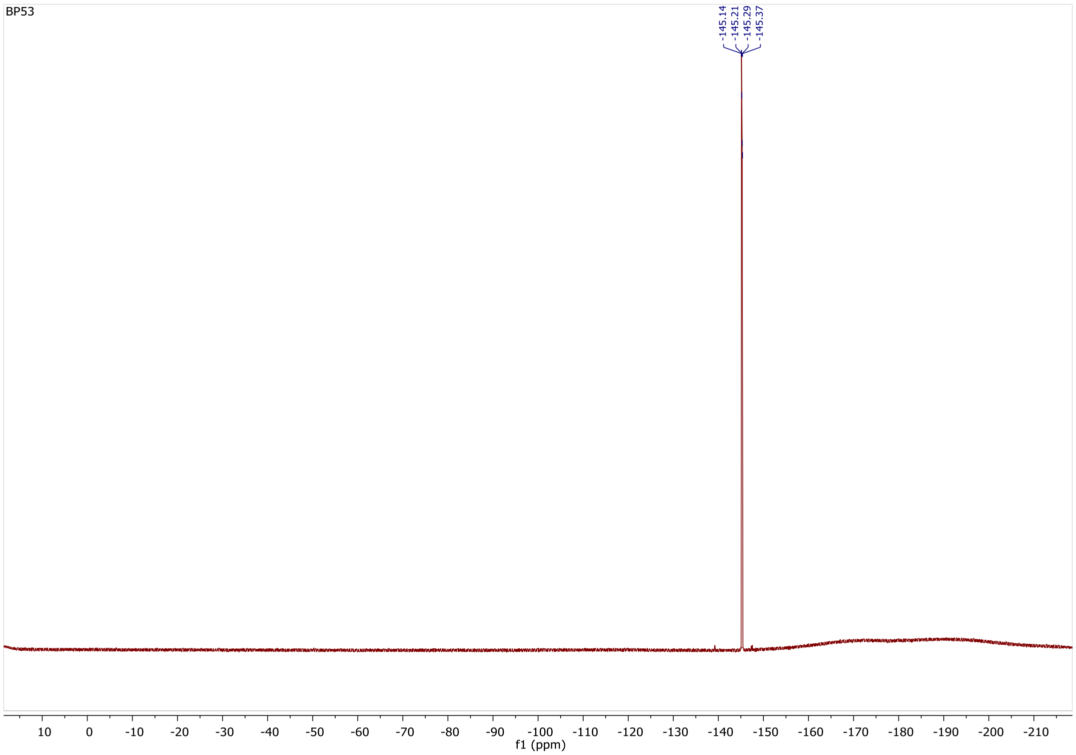

Figure S16. ^{19}F NMR spectrum of **BP-PH-OMe**.

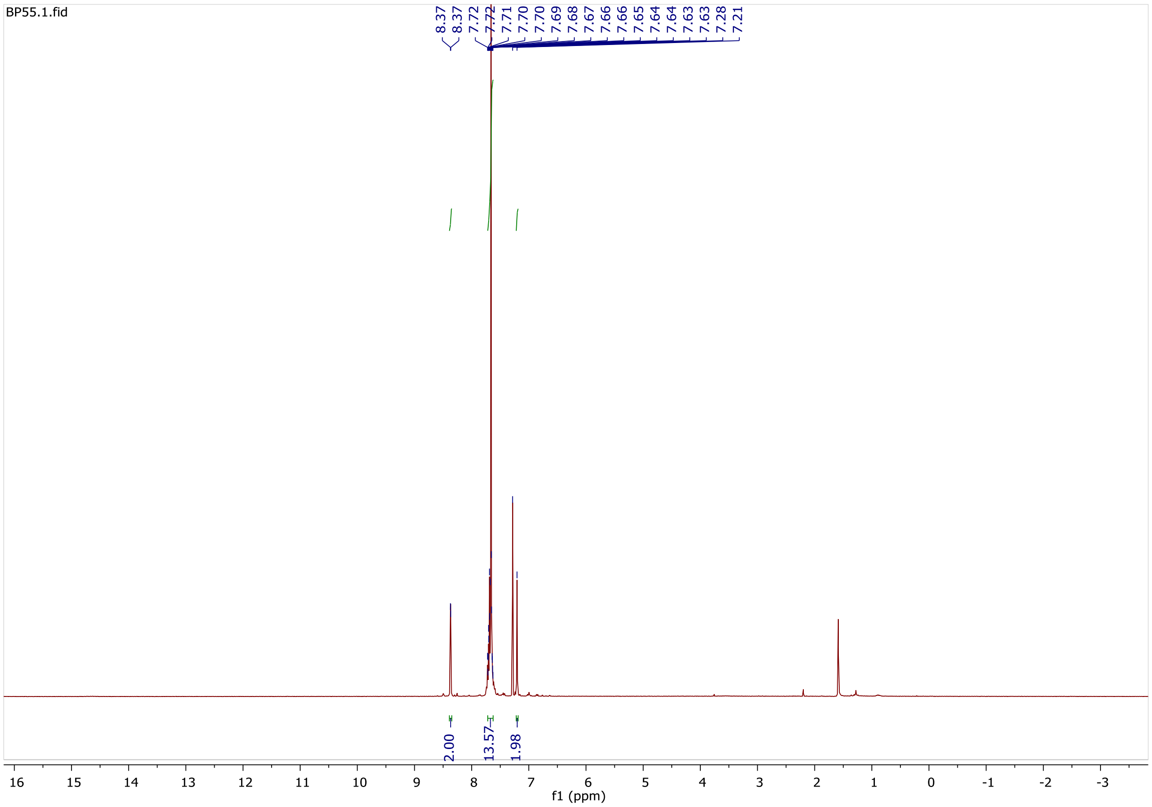

Figure S17. ^{1}H NMR spectrum of **BP-PH-CF$_3$**.

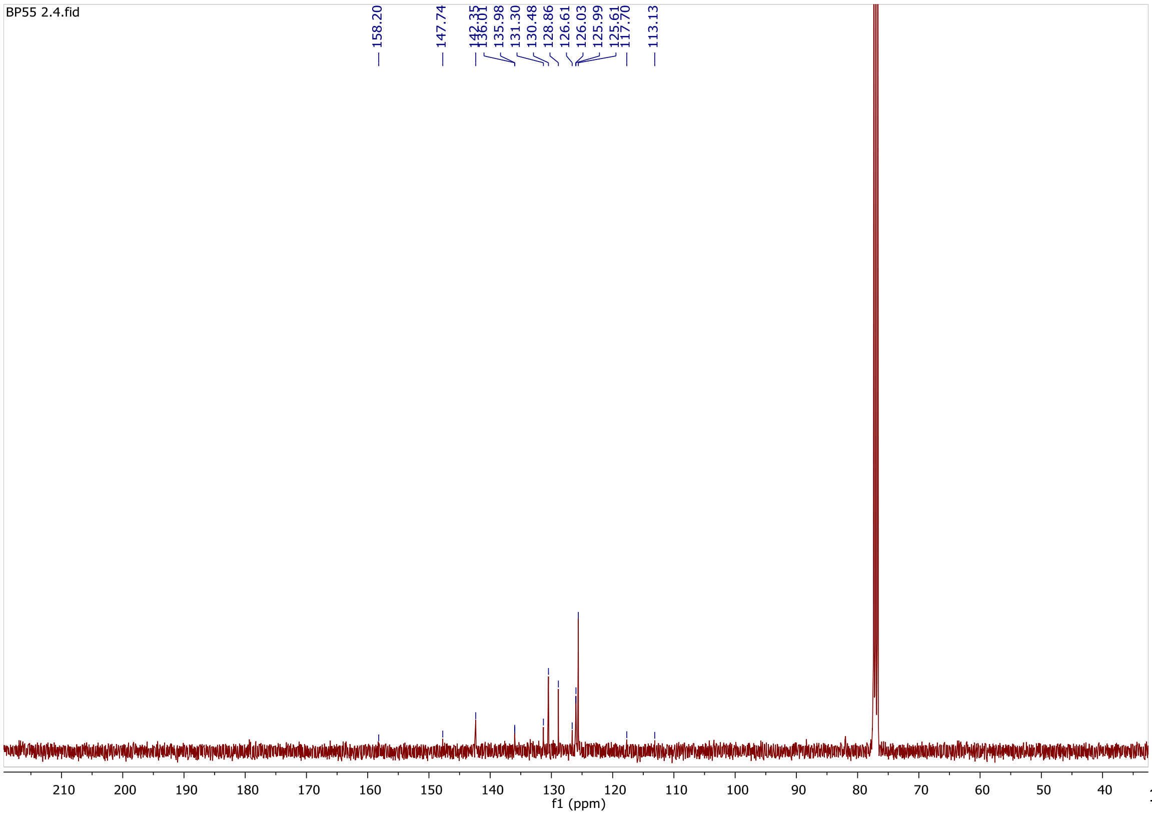

Figure S18. ^{3}C NMR spectrum of **BP-PH-CF$_3$**.

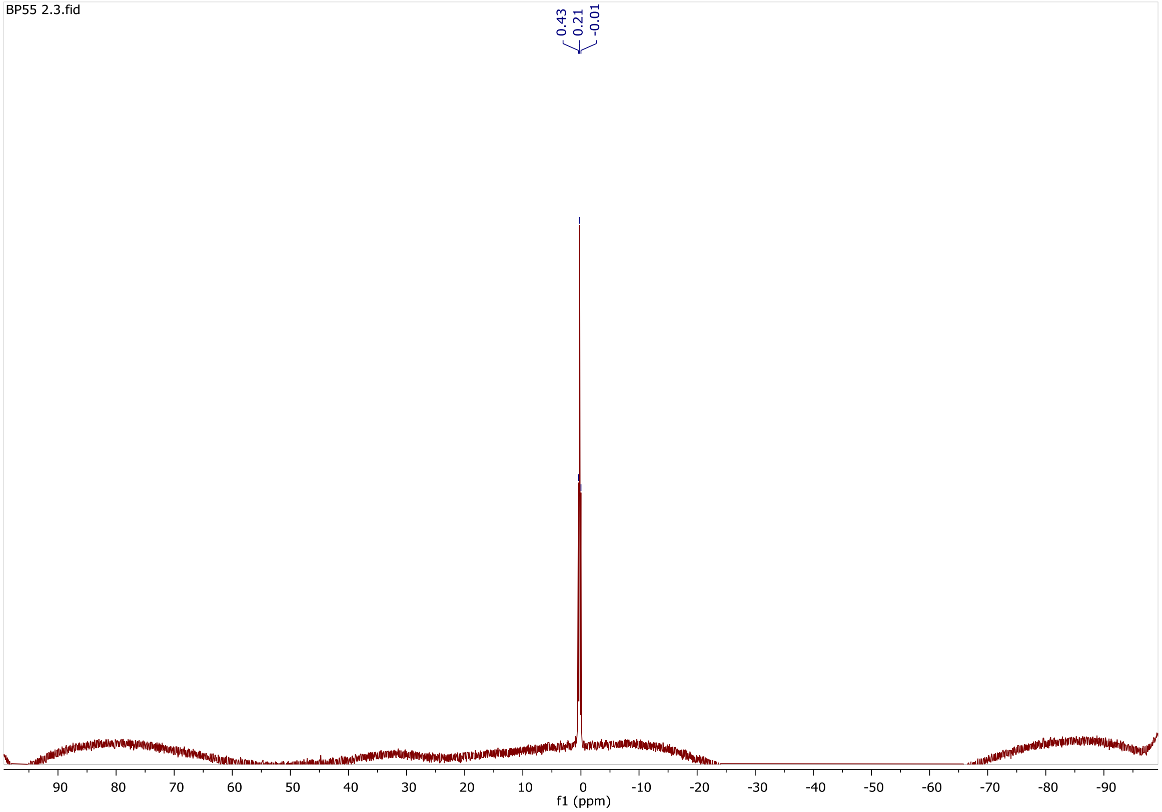

Figure S19. ^{11}B NMR spectrum of **BP-PH-CF$_3$**.

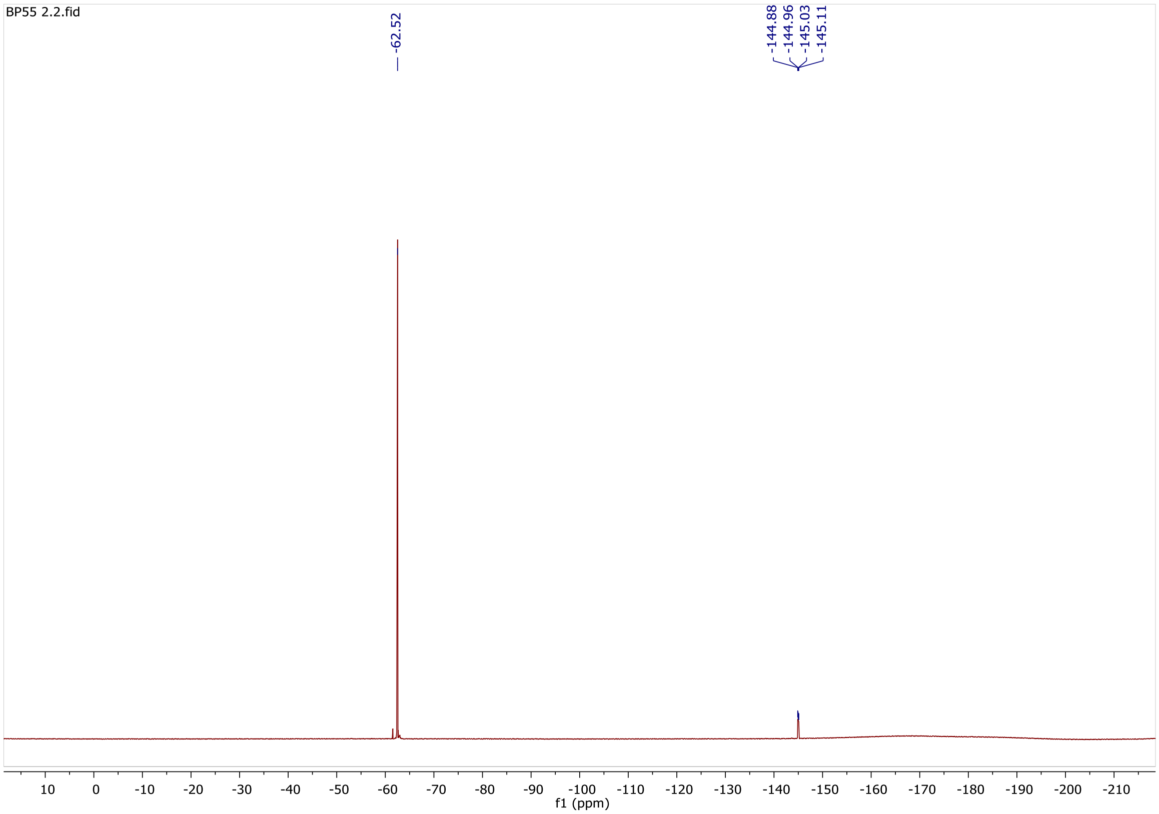

Figure S20. ^{19}F NMR spectrum of **BP-PH-CF$_3$**.

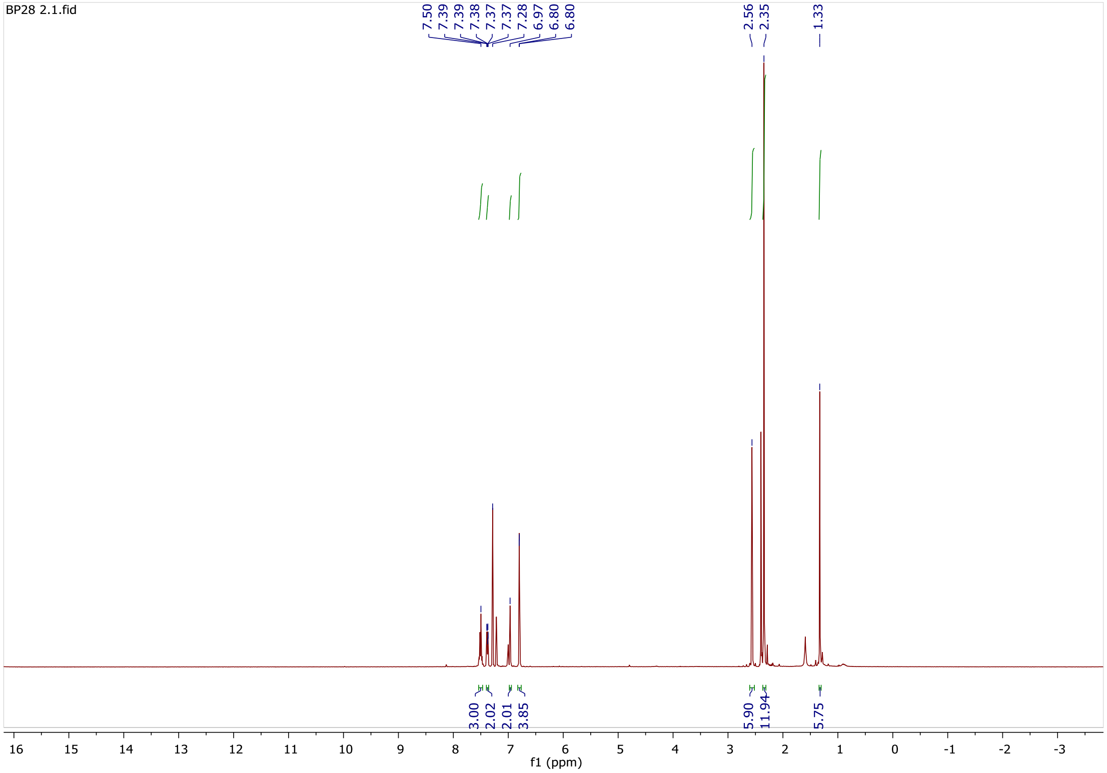

Figure S21. ^{1}H NMR spectrum of **BP-PH-8Me**.

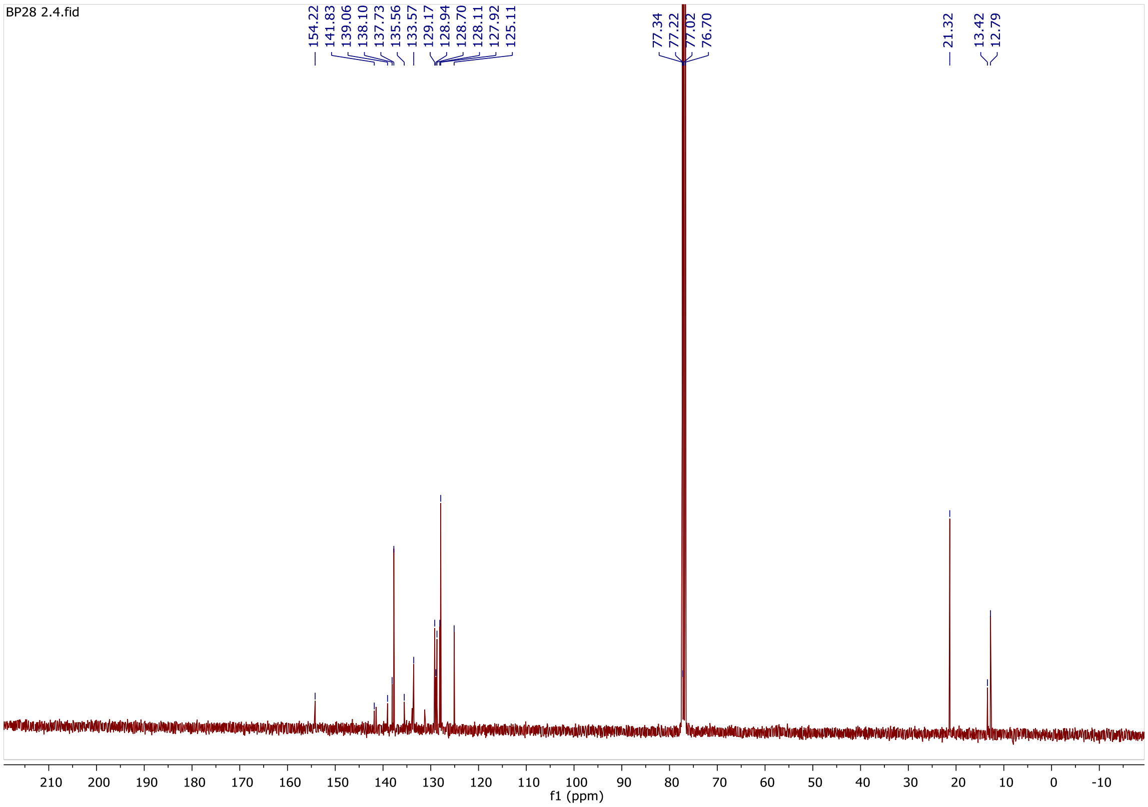

Figure S22. ^{13}C NMR spectrum of **BP-PH-8Me**.

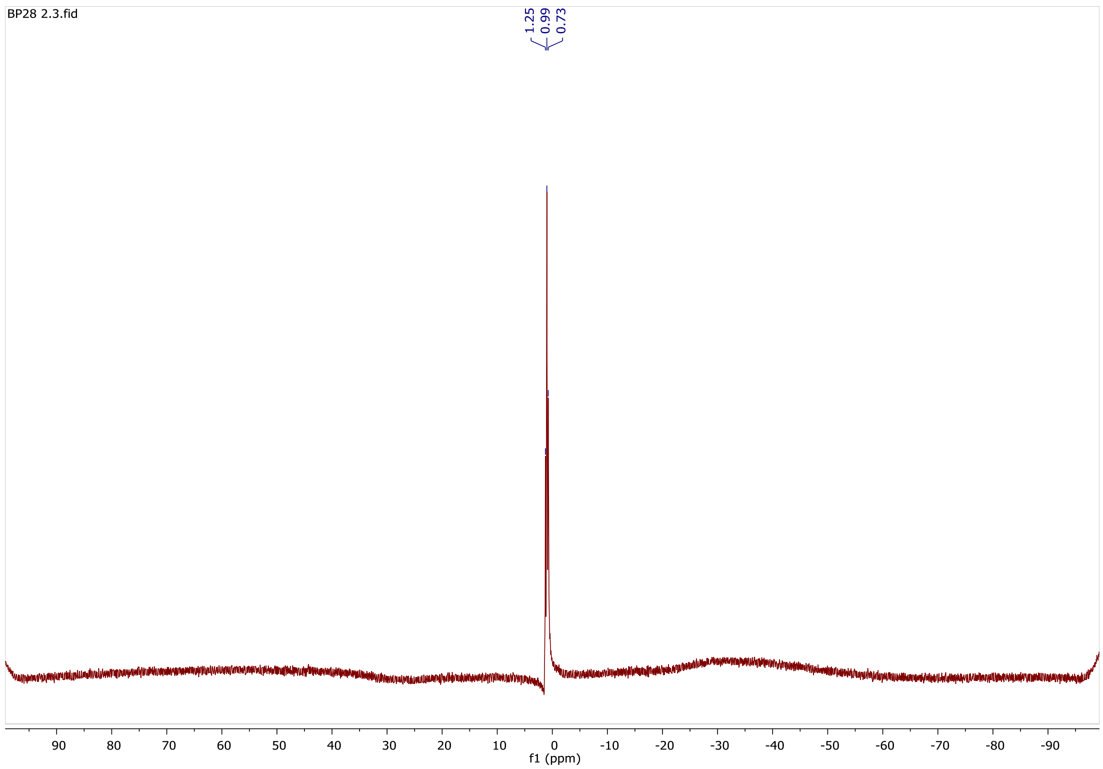

Figure S23. ^{11}B NMR spectrum of **BP-PH-8Me**.

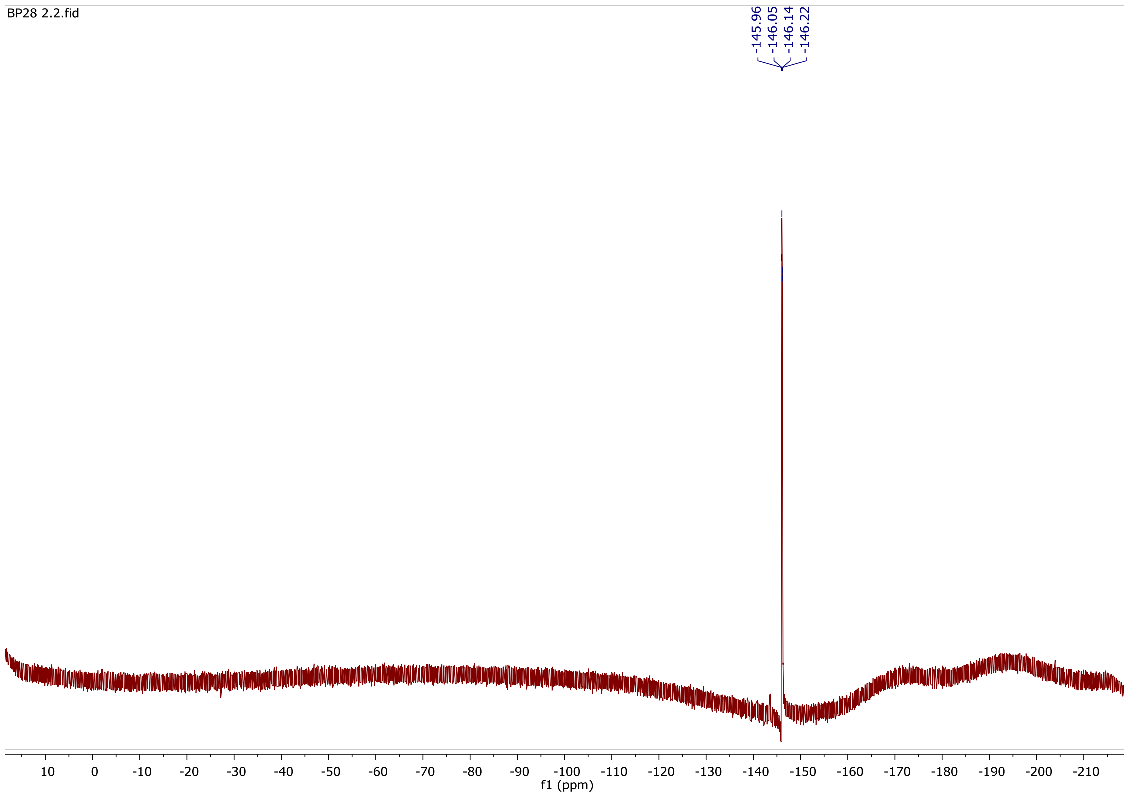

Figure S24. ^{19}F NMR spectrum of **BP-PH-8Me**.

References

1. Förster, T.; Hoffmann G. Effect of viscosity on the fluorescence quantum yield of some dye systems. *Zeitschrift fur physikalische chemie* **1971**, *75*, 63–76.
2. Kuimova, M. K. Mapping viscosity in cells using molecular rotors. *Physical Chemistry Chemical Physics*, **2012**, *14*, 12671–12686.
3. Lakowicz, J. Principles of Fluorescence Spectroscopy. *Springer*, **2006**, pp. 1-960
4. Becker, W. The bh TCSPC Handbook. *Scanning*, **2010**, pp. 1–566.
5. Toliautas, S.; Dodonova, J.; Žvirblis, A.; Čiplys, I.; Polita, A.; Devižis, A.; Tumkevičius, S.; Šulskus, J.; Vyšniauskas, A. Enhancing the Viscosity-Sensitive Range of a BODIPY Molecular Rotor by Two Orders of Magnitude. *Chemistry – A European Journal*, **2019**, *25*, 10342–10349.
6. Vázquez-Romero, A.; Kielland, N.; Arévalo, M. J.; Preciado, S.; Mellanby, R. J.; Feng, Y.; Lavilla, R.; Vendrell, M. Multicomponent Reactions for de Novo Synthesis of BODIPY Probes: In Vivo Imaging of Phagocytic Macrophages. *Journal of the American Chemical Society*, **2013**, *135*, 16018–16021.
7. Wu, W.; Guo, H.; Wu, W.; Ji, S.; Zhao, J. Organic Triplet Sensitizer Library Derived from a Single Chromophore (BODIPY) with Long-Lived Triplet Excited State for Triplet–Triplet Annihilation Based Upconversion. *The Journal of Organic Chemistry*, **2011**, *76*, 7056–7064.
